# Trends in unintentional injury death among post-9/11 Army Veterans who do and do not use Veteran Health Administration services

**DOI:** 10.1186/s40621-026-00666-5

**Published:** 2026-03-04

**Authors:** Rachel Sayko Adams, Jeri E. Forster, Claire A. Hoffmire, Jaimie L. Gradus, Trisha A. Hostetter, Mary Jo Larson, Alexandra L. Schneider, Alexandra A. Smith, Colin G. Walsh, Lisa A. Brenner

**Affiliations:** 1 Department of Health Law, Policy and Management, Boston University School of Public Health, 715 Albany Street, Boston, MA 02118 USA; 2 Veterans Affairs Rocky Mountain Mental Illness Research Education and Clinical Center, 1700 North Wheeling Street, Aurora, CO 80045 USA; 3 Department of Physical Medicine and Rehabilitation, University of Colorado, Anschutz Medical Campus, 12631 E 17th Ave, Aurora, CO 80045 USA; 4 Department of Epidemiology, Boston University School of Public Health, Boston University, 715 Albany Street, Boston, MA 02118 USA; 5 Institute for Behavioral Health, The Heller School for Social Policy and Management, Brandeis University, 415 South Street, Waltham, MA 02453 USA; 6 Departments of Biomedical Informatics, Medicine, and Psychiatry, Vanderbilt University Medical Center, 1211 Medical Center Drive, Nashville, TN 37232 USA; 7 Anschutz Medical Campus, Departments of PM&R, Psychiatry and Neurology, University of Colorado, 12631 E 17th Ave, Aurora, CO 80045 USA; 8 VA Brain Health Coordinating Center, Department of Veterans Affairs, Aurora, CO 80045 USA

**Keywords:** Unintentional injury death, Motor vehicle crash, Unintentional overdose, Military, Veterans, Deployment, Veterans Health Administration

## Abstract

**Background:**

It is unknown if unintentional injury death rates vary among Army Veterans who do and do not use the Veterans Health Administration (VHA). We estimated, and compared by VHA-use group, age-adjusted average annual unintentional injury death rates and time-dependent hazard rates (HRs) after separation.

**Methods:**

Data were from the Substance Use and Psychological Injury Combat Study, a longitudinal cohort of Army soldiers returning from an Afghanistan/Iraq deployment in FYs 2008–2014 using administrative data from the Military Health System, Veterans Health Administration and National Death Index. We selected soldiers who separated from active duty service by 9/30/2017 (*n* = 649,811). The exposure was time since separation, moderated by VHA-use group. The outcome was unintentional injury death (all, motor vehicle crash [MVC], overdose) through 2018.

**Results:**

The HR for all unintentional injury and MVC death for 0–2 years post-separation were higher for the no VHA-use group than the VHA-use group (e.g., MVC, year 0–1 HR: 75.1 (95% CI: 61.7, 88.4) vs. 16.4 (95% CI: 12.8, 20.0)). This changed to either no difference or higher risk for the VHA-use group as time since separation increased. For unintentional overdose, there was no difference in HRs between groups in 0–2 years post-separation; risk was higher for the VHA-use group for the remainder of follow-up (e.g., no VHA use, years 3–5 HR: 8.7 (95% CI: 5.1, 12.3) vs. 28.9 (95% CI: 25.2, 32.5) for VHA use group).

**Conclusions:**

The risk for unintentional MVC and overdose death since separation differed based on use of VHA services among post-9/11 Veterans. Intervention strategies that address differences in time since separation and whether Veterans use the VHA or not may be required.

**Supplementary Information:**

The online version contains supplementary material available at 10.1186/s40621-026-00666-5.

## Background

Unintentional injury deaths including those by motor vehicle crashes (MVC) and overdose are the most common cause of death among post-9/11 military Veterans [1–4]. While studies have compared unintentional injury death rates among post-9/11 Veterans by military separation status [1] and deployment history, [4] it is unknown if such rates vary among Veterans who do and do not use the Veterans Health Administration (i.e., VHA-use group).

Veterans who separated from military service after deploying to Afghanistan or Iraq were eligible for at least 10 years of care in the VHA, with a few exceptions (those with other-than-honorable service designations) [5]. However, prior studies have shown that a significant portion of post-9/11 Veterans who were eligible did not use the VHA, and importantly there were significant differences between these two groups in terms of psychological and physical health needs [6, 7]. Therefore, it is important to understand if trends in unintentional injury death rates vary among Veterans who do and do not use the VHA in order to help inform development of tailored intervention strategies to prevent deaths among members of these two groups. To address these gaps in knowledge, this study used a population-based cohort of 649,811 Veterans who separated from active duty after an Afghanistan/Iraq deployment to estimate: (1) age-adjusted average annual unintentional injury death rates (all, MVAC, overdose) and compared by VHA-use group; and (2) time-dependent hazard rates and trends for unintentional injury death after military service and compared by VHA-use group.

## Methods

Data were from the Substance Use and Psychological Injury Combat Study (SUPIC), an observational, population-based, longitudinal cohort study of 860,930 soldiers returning from an Afghanistan/Iraq deployment of ≤ 5 years ending in FYs 2008–2014. The SUPIC population-based cohort represented 99.5% of all Army soldiers returning from an Afghanistan/Iraq deployment during these study years, after exclusions for missing data [8]. Deployment data was from the Contingency Tracking System, used to identify the index Afghanistan/Iraq deployment (i.e., first deployment ending during the study window). Demographic data was from the Defense Enrollment Eligibility Records System. Mortality data was from the VA/DoD Mortality Data Repository (MDR), containing National Death Index (NDI) data of death records from state vital statistics offices. VHA utilization information came from the Corporate Data Warehouse.

The last month of Military Health System (MHS) eligibility based on active duty status was used as a proxy for separation from active duty. Due to MHS data availability, the sample was limited to those who separated from active duty by 9/30/2017, approximately 76% of the SUPIC cohort (*n* = 650,072). Given these data are updated monthly, we excluded soldiers who died within 30 days after separation to reduce the possibility that the death occurred because of an event during active duty (*n* = 265, 0.04%). The analytic sample was *n* = 649,811.

### Measures

VHA-use was determined by the presence of at least one encounter, inpatient stay or encounter paid for by VA from 90 days prior to separation from active duty through December 31, 2018. Unintentional injury deaths (i.e., all; MVC; overdose) were classified using underlying cause of death ICD-10 codes (see Additional File-1 ) [3]. We report age group at separation from active duty.

### Statistical Analysis

VHA-use groups were summarized by age group and the following causes of death: (1) all unintentional injury; (2) unintentional MVC; and (3) unintentional overdose, using frequencies. The follow-up period, October 1, 2007–December 31, 2018, encompassed up to 11.3 years, depending on active duty separation date. Direct age-adjusted average annual mortality rates were calculated, by cause of death, per 100,000 person-years over the follow-up period. The 2000 US population was used for direct standardization, [9] using age categories 18–24, 25–29, 30–34, 35–39, and 40+, unless otherwise noted. Rates based on < 16 events were considered unreliable and those based on < 10 were suppressed [10]. Rate ratios (RRs) were computed to compare age-adjusted rates. All estimates reported include 95% confidence intervals (CIs).

Time dependent hazard rates (based on time since separation from active duty), per 100,000 alive at the beginning of each time interval, were calculated using the life-table method by cause of death and VHA-use group. Time intervals were measured in years, from time of separation from active duty to death or the end of the study, and interval lengths (1–6 years) were chosen to minimize estimate suppression and unreliable estimates. Analyses were performed in SAS v9.4.

## Results

Within our sample, 75.2% (*n* = 488,362) of Veterans used VHA for healthcare from separation through the end of the study time period (12/31/2018) or death, whichever came first (Table 1). The average time from separation from active duty to the end of the study or death was slightly longer for Veterans who used VHA (6.78 vs. 6.31 years). Veterans who used VHA were older compared to those who did not. Among the 2,453 unintentional injury deaths, 44.8% were MVCs and 41.0% were overdoses; 75.9% were among VHA users and 24.1% among non-VHA users.

**Table 1:** Demographic Characteristics and Number of Unintentional Injury Deaths,^a^ by VHA-Use Group

Characteristic (sample size)	Overall (*N* = 649,811, 100%)	No-VHA-Use (*n* = 161,449, 24.8%)	VHA-Use (*n* = 488,362, 75.2%)
	*n* (%) or mean (SD)	*n* (%) or mean (SD)	*n* (%) or mean (SD)
Age at Separation from Active Duty			
18–24	184,720 (28.43%)	50,370 (31.20%)	134,350 (27.51%)
25–29	183,288 (28.21%)	47,663 (29.52%)	135,625 (27.77%)
30–34	92,479 (14.23%)	22,284 (13.80%)	70,195 (14.37%)
35–39	62,680 (9.65%)	14,026 (8.69%)	48,654 (9.96%)
40+	126,644 (19.49%)	27,106 (16.79%)	99,538 (20.38%)
Time Since Active Duty Separation to End of Study or Death (years)	6.67 (2.5)	6.31 (2.5)	6.78 (2.4)
Deaths			
Number of Unintentional Injury Deaths (all)	2453 (0.38%)	592 (0.37%)	1861 (0.38%)
Number of Unintentional Motor Vehicle Crash Deaths	1099 (0.17%)	362 (0.22%)	737 (0.15%)
Number of Unintentional Overdose Deaths	1005 (0.15%)	114 (0.07%)	891 (0.18%)

VHA = Veterans Health Administration; Number of Unintentional Injury Deaths (all) includes MVC, Overdose and all other types of Unintentional Injury Death

^a^ From Active Duty Separation to 12/31/2018

No significant difference was identified for the overall unintentional injury death rate by VHA-use group. Unintentional injury death rates revealed lower MVC death rates (RR = 0.74, *p* = 0.004) and higher rates of overdose deaths (RR = 3.05, *p* < 0.0001) for the VHA-use group compared to the non-VHA-use group (Table 2).

**Table 2:** Age-Adjusted Unintentional Injury Death Rates per 100,000 person years with 95% CIs

-	No-VHA-Use (*n* = 161,449)	VHA-Use (*n* = 488,362)	Rate Ratio (95% CI)	*p*-value
All Unintentional Injury Deaths	42.88 (37.23, 49.34)	41.63 (38.87, 44.58)	0.97 (0.83, 1.13)	0.70
Unintentional MVC Deaths	24.24 (20.22, 29.07)	17.95 (16.08, 20.03)	0.74 (0.60, 0.91)	0.004
Unintentional Overdose Deaths	5.75 (4.26, 7.84)	17.50 (15.93, 19.23)	3.05 (2.27, 4.08)	< 0.0001

VHA = Veterans Health Administration; Number of Unintentional Injury Deaths (all) includes MVC, Overdose and all other types of Unintentional Injury Death

^a^ From Active Duty Separation to 12/31/2018

**Fig. 1:**
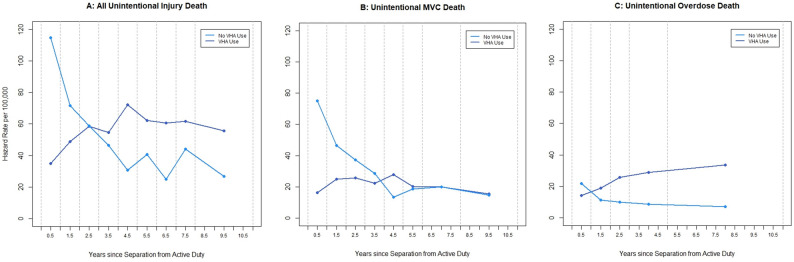
Hazard rates for unintentional injury death since separation from active duty, by VHA use group. All unintentional injury death, unintentional MVC death, and unintentional overdose death hazard rates with trend lines, by use of Veteran Health Administration ServicesCaption: Hazard rates and 95% confidence intervals are in the Additional File-2 .

The risk of death due to unintentional MVC and overdose changed over time since separation from active duty by VHA-use group (Fig. 1; Additional File-2). Hazard rates for all unintentional injury deaths were significantly higher (i.e., 95% CIs do not overlap) for the non-VHA-use group in the 1st -2nd years after separation, were not different in the 3rd -4th years, then switched directions to become significantly higher for the VHA-use group in the 5th -7th years, nonsignificant in the 8th year, and significantly higher again thereafter. Hazard rates for unintentional MVC were significantly higher in the first two years following separation among the non-VHA-use group, were not significantly different in the 3rd -4th years, switched directions to become significantly higher in the 5th year for the VHA-use group, and were nonsignificant thereafter. Hazard rates for unintentional overdose deaths in the first two years post-separation were not different by VHA-use group, but were generally significantly higher thereafter for the VHA-use group.

## Discussion

This study of post-9/11 Veterans who had one or more deployments revealed that unintentional MVC death rates were higher among the non-VHA-use group, while unintentional overdose death rates were generally higher among the VHA-use group. Further, our findings demonstrate that the trajectory of risk for unintentional MVC and overdose deaths differed by VHA-use groups. Risk for unintentional MVC deaths was highest in the immediate years following active duty separation among the non-VHA-use group; however, around 4–5 years post separation, risk of unintentional MVC deaths was significantly higher for the VHA-use group. Conversely, unintentional overdose deaths were lowest in the first few years following separation among the VHA-use group, followed by an increase in rate, which remained elevated through the end of the study period (i.e., up to 11 years).

Our findings add to the growing literature that risk for unintentional MVC death is high following military separation [11, 12]. We contribute new knowledge that risk of unintentional MVC death is highest among Veterans who do not use the VHA in the first few years following active duty separation. More research is needed to understand if Veterans with excessive alcohol use or other risk factors not accounted for here (e.g., military separation for substance use) are less likely to use VHA in the first few years after separating from service. It is logistically complex to develop interventions to support Veterans who do not use the VHA due to a lack of systematic contact with the integrated VHA. Therefore, developing interventions to decrease risky driving are needed before separation and efforts should be developed to continue outreach to Veterans who do not use the VHA through the next several years. Interventions should incorporate potential contributors to risky driving (e.g., risky drinking, driving while under the influence, aggressive driving) [3, 11–13].

Expanding upon studies that have examined unintentional overdose deaths among Veterans using VHA, [14, 15] we found that unintentional overdose death rates were higher among Veterans using the VHA compared to those not using VHA starting around 3 years after active duty separation, remaining significantly elevated thereafter. While our study did not examine the portion of unintentional overdose deaths that were opioid-related, one possible explanation for these findings is that post-9/11 Veterans receiving care in the VHA may be more likely to receive pain management inclusive of prescription opioids which may, in turn, contribute to unintentional overdose deaths via increased access to lethal means. Prior research revealed that the majority of Army Veterans identified with chronic pain while in military service used VHA after military separation, and that among those who starting using VHA, approximately 50% met criteria for chronic pain during their first year in VHA, and 41% of those Veterans received prescription opioids in VHA that year [7]. That being said, VHA has implemented enterprise-wide strategies (e.g., Academic Detailing Services) to address opioid safety and promote opioid stewardship [16]. Many of these strategies are contingent upon the VHA’s integrated system of care and electronic medical record, and as such would be difficult to implement in non-VA settings. Alternatively, these findings may reflect a higher likelihood for Veterans living with substance use disorders to seek VHA care, putting the VHA user population at greater baseline risk for unintentional overdose deaths. While additional research is needed to elucidate drivers of the observed differences across VHA-use groups in this study, including but not limited to differentiating opioid vs. non-opioid unintentional overdoses, present findings indicate that it is critical for VHA to continue prioritizing programs and policies to reduce non-fatal and fatal drug overdoses [17].

Study limitations include possible misclassification of cause of death. Estimates of time of separation from active duty may be incorrect for some Veterans, as we did not have exact dates for our proxy of the end of active Military Health System eligibility. Study results may not be generalizable to post 9/11 Veterans from other service branches, Army soldiers who returned from Afghanistan/Iraq during FYs outside of our study window, or SUPIC cohort soldiers who did not leave military service by the end of 2017. We did not have information about whether military separations were honorable or other than honorable, or specific VHA eligibility information; however, we note that the majority of SUPIC cohort members who separated from active duty should have been eligible for up to 10 years of VHA care due to their Afghanistan/Iraq deployment [5]. Future studies should examine how sociodemographic characteristics (e.g., sex recorded in the medical record), military history characteristics (e.g., reason for military separation, number of deployments), or clinical characteristics during military service (e.g., mental health or traumatic brain injury diagnoses) were associated with risk for unintentional injury death since military separation within the VHA and non-VHA groups, as well as timing of VHA service use post-active duty separation. We defined the VHA use group by the presence of at least one encounter, inpatient stay or encounter paid for by VA; future studies should examine if timing, intensity, or type of treatment in the VHA following military separation is associated with varying risk for unintentional injury death following military separation.

## Conclusions

Risk of unintentional MVC and overdose deaths among post-9/11 Veterans following active duty separation differed by use of VHA services. Higher risk of unintentional MVC deaths among Veterans who did not use VHA services proximal to active duty separation suggests that military and transition support services may be most effective at reducing such deaths. Conversely, higher risk of unintentional overdose deaths among Veterans using VHA services occurring distally from active duty separation points to the importance of interventions within VHA to curb risk.

## Supplementary Information

Additional File-1

Additional File-2

## Data Availability

The Defense Health Agency’s Privacy and Civil Liberties Office provided access to Department of Defense (DoD) data and the VA/DOD Mortality Data Repository (MDR) provided access to National Death Index data. The datasets generated and analyzed during the current study are not publicly available according to our Data Sharing Agreements and are governed by the Defense Health Agency and Veterans Health Affairs, and the MDR, respectively.

## Abbreviations

VHA: Veterans Health Administration
HR: Hazard Rate
FY: Fiscal Year
MVC: Motor Vehicle Crash
CI: Confidence Interval
SUPIC: Substance Use and Psychological Injury Combat Study
MHS: Military Health System
VA: Veterans Administration
RR: Rate Ratios
