# Supplementary material for: Trends in unintentional injury death among post-9/11 Army Veterans who do and do not use Veteran Health Administration services
Source: Inj Epidemiol. 2026 Mar 4;13:27. doi: 10.1186/s40621-026-00666-5 (PMC13069699; doi:10.1186/s40621-026-00666-5)
Supplement: Supplementary file 1 — Additional File-1 [file 40621_2026_666_MOESM1_ESM.docx]

**Supplemental Table 1: Unintentional Injury Death ICD-10 Codes**

| **ICD-10 Code** | **ICD-10 Description** | **Unintentional motor vehicle crash death** | **Unintentional overdose death** |
| --- | --- | --- | --- |
| V01.0 | Pedestrian Injured In Collision With Pedal Cycle In Nontraffic Accident | No | No |
| V01.1 | Pedestrian Injured In Collision With Pedal Cycle In Traffic Accident | No | No |
| V01.9 | Pedestrian Injured In Collision With Pedal Cycle, Unspecified Whether Traffic Or Nontraffic Accident | No | No |
| V02.0 | Pedestrian Injured In Collision With Two- Or Three-Wheeled Motor Vehicle In Nontraffic Accident | Yes | No |
| V02.1 | Pedestrian Injured In Collision With Two- Or Three-Wheeled Motor Vehicle In Traffic Accident | Yes | No |
| V02.9 | Pedestrian Injured In Collision With Two- Or Three-Wheeled Motor Vehicle, Unspecified Whether Traffic Or Nontraffic Accident | Yes | No |
| V03.0 | Pedestrian Injured In Collision With Car, Pick-Up Truck Or Van In Nontraffic Accident | Yes | No |
| V03.1 | Pedestrian Injured In Collision With Car, Pick-Up Truck Or Van In Traffic Accident | Yes | No |
| V03.9 | Pedestrian Injured In Collision With Car, Pick-Up Truck Or Van, Unspecified Whether Traffic Or Nontraffic Accident | Yes | No |
| V04.0 | Pedestrian Injured In Collision With Heavy Transport Vehicle Or Bus In Nontraffic Accident | Yes | No |
| V04.1 | Pedestrian Injured In Collision With Heavy Transport Vehicle Or Bus In Traffic Accident | Yes | No |
| V04.9 | Pedestrian Injured In Collision With Heavy Transport Vehicle Or Bus, Unspecified Whether Traffic Or Nontraffic Accident | Yes | No |
| V05.0 | Pedestrian Injured In Collision With Railway Train Or Railway Vehicle In Nontraffic Accident | No | No |
| V05.1 | Pedestrian Injured In Collision With Railway Train Or Railway Vehicle In Traffic Accident | No | No |
| V05.9 | Pedestrian Injured In Collision With Railway Train Or Railway Vehicle, Unspecified Whether Traffic Or Nontraffic Accident | No | No |
| V06.0 | Pedestrian Injured In Collision With Other Nonmotor Vehicle In Nontraffic Accident | No | No |
| V06.1 | Pedestrian Injured In Collision With Other Nonmotor Vehicle In Traffic Accident | No | No |
| V06.9 | Pedestrian Injured In Collision With Other Nonmotor Vehicle, Unspecified Whether Traffic Or Nontraffic Accident | No | No |
| V09.0 | Pedestrian Injured In Nontraffic Accident Involving Other And Unspecified Motor Vehicles | Yes | No |
| V09.1 | Pedestrian Injured In Unspecified Nontraffic Accident | No | No |
| V09.2 | Pedestrian Injured In Traffic Accident Involving Other And Unspecified Motor Vehicles | Yes | No |
| V09.3 | Pedestrian Injured In Unspecified Traffic Accident | No | No |
| V09.9 | Pedestrian Injured In Unspecified Transport Accident | No | No |
| V10.0 | Pedal Cycle Driver Injured In Collision With Pedestrian Or Animal In Nontraffic Accident | No | No |
| V10.1 | Pedal Cycle Passenger Injured In Collision With Pedestrian Or Animal In Nontraffic Accident | No | No |
| V10.2 | Unspecified Pedal Cyclist Injured In Collision With Pedestrian Or Animal In Nontraffic Accident | No | No |
| V10.3 | Person Boarding Or Alighting A Pedal Cycle Injured In Collision With Pedestrian Or Animal | No | No |
| V10.4 | Pedal Cycle Driver Injured In Collision With Pedestrian Or Animal In Traffic Accident | No | No |
| V10.5 | Pedal Cycle Passenger Injured In Collision With Pedestrian Or Animal In Traffic Accident | No | No |
| V10.9 | Unspecified Pedal Cyclist Injured In Collision With Pedestrian Or Animal In Traffic Accident | No | No |
| V11.0 | Pedal Cycle Driver Injured In Collision With Other Pedal Cycle In Nontraffic Accident | No | No |
| V11.1 | Pedal Cycle Passenger Injured In Collision With Other Pedal Cycle In Nontraffic Accident | No | No |
| V11.2 | Unspecified Pedal Cyclist Injured In Collision With Other Pedal Cycle In Nontraffic Accident | No | No |
| V11.3 | Person Boarding Or Alighting A Pedal Cycle Injured In Collision With Other Pedal Cycle | No | No |
| V11.4 | Pedal Cycle Driver Injured In Collision With Other Pedal Cycle In Traffic Accident | No | No |
| V11.5 | Pedal Cycle Passenger Injured In Collision With Other Pedal Cycle In Traffic Accident | No | No |
| V11.9 | Unspecified Pedal Cyclist Injured In Collision With Other Pedal Cycle In Traffic Accident | No | No |
| V12.0 | Pedal Cycle Driver Injured In Collision With Two- Or Three-Wheeled Motor Vehicle In Nontraffic Accident | Yes | No |
| V12.1 | Pedal Cycle Passenger Injured In Collision With Two- Or Three-Wheeled Motor Vehicle In Nontraffic Accident | Yes | No |
| V12.2 | Unspecified Pedal Cyclist Injured In Collision With Two- Or Three-Wheeled Motor Vehicle In Nontraffic Accident | Yes | No |
| V12.3 | Person Boarding Or Alighting A Pedal Cycle Injured In Collision With Two- Or Three-Wheeled Motor Vehicle | Yes | No |
| V12.4 | Pedal Cycle Driver Injured In Collision With Two- Or Three-Wheeled Motor Vehicle In Traffic Accident | Yes | No |
| V12.5 | Pedal Cycle Passenger Injured In Collision With Two- Or Three-Wheeled Motor Vehicle In Traffic Accident | Yes | No |
| V12.9 | Unspecified Pedal Cyclist Injured In Collision With Two- Or Three-Wheeled Motor Vehicle In Traffic Accident | Yes | No |
| V13.0 | Pedal Cycle Driver Injured In Collision With Car, Pick-Up Truck Or Van In Nontraffic Accident | Yes | No |
| V13.1 | Pedal Cycle Passenger Injured In Collision With Car, Pick-Up Truck Or Van In Nontraffic Accident | Yes | No |
| V13.2 | Unspecified Pedal Cyclist Injured In Collision With Car, Pick-Up Truck Or Van In Nontraffic Accident | Yes | No |
| V13.3 | Person Boarding Or Alighting A Pedal Cycle Injured In Collision With Car, Pick-Up Truck Or Van | Yes | No |
| V13.4 | Pedal Cycle Driver Injured In Collision With Car, Pick-Up Truck Or Van In Traffic Accident | Yes | No |
| V13.5 | Pedal Cycle Passenger Injured In Collision With Car, Pick-Up Truck Or Van In Traffic Accident | Yes | No |
| V13.9 | Unspecified Pedal Cyclist Injured In Collision With Car, Pick-Up Truck Or Van In Traffic Accident | Yes | No |
| V14.0 | Pedal Cycle Driver Injured In Collision With Heavy Transport Vehicle Or Bus In Nontraffic Accident | Yes | No |
| V14.1 | Pedal Cycle Passenger Injured In Collision With Heavy Transport Vehicle Or Bus In Nontraffic Accident | Yes | No |
| V14.2 | Unspecified Pedal Cyclist Injured In Collision With Heavy Transport Vehicle Or Bus In Nontraffic Accident | Yes | No |
| V14.3 | Person Boarding Or Alighting A Pedal Cycle Injured In Collision With Heavy Transport Vehicle Or Bus | Yes | No |
| V14.4 | Pedal Cycle Driver Injured In Collision With Heavy Transport Vehicle Or Bus In Traffic Accident | Yes | No |
| V14.5 | Pedal Cycle Passenger Injured In Collision With Heavy Transport Vehicle Or Bus In Traffic Accident | Yes | No |
| V14.9 | Unspecified Pedal Cyclist Injured In Collision With Heavy Transport Vehicle Or Bus In Traffic Accident | Yes | No |
| V15.0 | Pedal Cycle Driver Injured In Collision With Railway Train Or Railway Vehicle In Nontraffic Accident | No | No |
| V15.1 | Pedal Cycle Passenger Injured In Collision With Railway Train Or Railway Vehicle In Nontraffic Accident | No | No |
| V15.2 | Unspecified Pedal Cyclist Injured In Collision With Railway Train Or Railway Vehicle In Nontraffic Accident | No | No |
| V15.3 | Person Boarding Or Alighting A Pedal Cycle Injured In Collision With Railway Train Or Railway Vehicle | No | No |
| V15.4 | Pedal Cycle Driver Injured In Collision With Railway Train Or Railway Vehicle In Traffic Accident | No | No |
| V15.5 | Pedal Cycle Passenger Injured In Collision With Railway Train Or Railway Vehicle In Traffic Accident | No | No |
| V15.9 | Unspecified Pedal Cyclist Injured In Collision With Railway Train Or Railway Vehicle In Traffic Accident | No | No |
| V16.0 | Pedal Cycle Driver Injured In Collision With Other Nonmotor Vehicle In Nontraffic Accident | No | No |
| V16.1 | Pedal Cycle Passenger Injured In Collision With Other Nonmotor Vehicle In Nontraffic Accident | No | No |
| V16.2 | Unspecified Pedal Cyclist Injured In Collision With Other Nonmotor Vehicle In Nontraffic Accident | No | No |
| V16.3 | Person Boarding Or Alighting A Pedal Cycle Injured In Collision With Other Nonmotor Vehicle In Nontraffic Accident | No | No |
| V16.4 | Pedal Cycle Driver Injured In Collision With Other Nonmotor Vehicle In Traffic Accident | No | No |
| V16.5 | Pedal Cycle Passenger Injured In Collision With Other Nonmotor Vehicle In Traffic Accident | No | No |
| V16.9 | Unspecified Pedal Cyclist Injured In Collision With Other Nonmotor Vehicle In Traffic Accident | No | No |
| V17.0 | Pedal Cycle Driver Injured In Collision With Fixed Or Stationary Object In Nontraffic Accident | No | No |
| V17.1 | Pedal Cycle Passenger Injured In Collision With Fixed Or Stationary Object In Nontraffic Accident | No | No |
| V17.2 | Unspecified Pedal Cyclist Injured In Collision With Fixed Or Stationary Object In Nontraffic Accident | No | No |
| V17.3 | Person Boarding Or Alighting A Pedal Cycle Injured In Collision With Fixed Or Stationary Object | No | No |
| V17.4 | Pedal Cycle Driver Injured In Collision With Fixed Or Stationary Object In Traffic Accident | No | No |
| V17.5 | Pedal Cycle Passenger Injured In Collision With Fixed Or Stationary Object In Traffic Accident | No | No |
| V17.9 | Unspecified Pedal Cyclist Injured In Collision With Fixed Or Stationary Object In Traffic Accident | No | No |
| V18.0 | Pedal Cycle Driver Injured In Noncollision Transport Accident In Nontraffic Accident | No | No |
| V18.1 | Pedal Cycle Passenger Injured In Noncollision Transport Accident In Nontraffic Accident | No | No |
| V18.2 | Unspecified Pedal Cyclist Injured In Noncollision Transport Accident In Nontraffic Accident | No | No |
| V18.3 | Person Boarding Or Alighting A Pedal Cycle Injured In Noncollision Transport Accident | No | No |
| V18.4 | Pedal Cycle Driver Injured In Noncollision Transport Accident In Traffic Accident | No | No |
| V18.5 | Pedal Cycle Passenger Injured In Noncollision Transport Accident In Traffic Accident | No | No |
| V18.9 | Unspecified Pedal Cyclist Injured In Noncollision Transport Accident In Traffic Accident | No | No |
| V19.0 | Pedal Cycle Driver Injured In Collision With Other And Unspecified Motor Vehicles In Nontraffic Accident | Yes | No |
| V19.1 | Pedal Cycle Passenger Injured In Collision With Other And Unspecified Motor Vehicles In Nontraffic Accident | Yes | No |
| V19.2 | Unspecified Pedal Cyclist Injured In Collision With Other And Unspecified Motor Vehicles In Nontraffic Accident | Yes | No |
| V19.3 | Pedal Cyclist (Driver) (Passenger) Injured In Unspecified Nontraffic Accident | No | No |
| V19.4 | Pedal Cycle Driver Injured In Collision With Other And Unspecified Motor Vehicles In Traffic Accident | Yes | No |
| V19.5 | Pedal Cycle Passenger Injured In Collision With Other And Unspecified Motor Vehicles In Traffic Accident | Yes | No |
| V19.6 | Unspecified Pedal Cyclist Injured In Collision With Other And Unspecified Motor Vehicles In Traffic Accident | Yes | No |
| V19.8 | Pedal Cyclist (Driver) (Passenger) Injured In Other Specified Transport Accidents | No | No |
| V19.9 | Pedal Cyclist (Driver) (Passenger) Injured In Unspecified Traffic Accident | No | No |
| V20.0 | Motorcycle Driver Injured In Collision With Pedestrian Or Animal In Nontraffic Accident | Yes | No |
| V20.1 | Motorcycle Passenger Injured In Collision With Pedestrian Or Animal In Nontraffic Accident | Yes | No |
| V20.2 | Unspecified Motorcycle Rider Injured In Collision With Pedestrian Or Animal In Nontraffic Accident | Yes | No |
| V20.3 | Person Boarding Or Alighting A Motorcycle Injured In Collision With Pedestrian Or Animal | Yes | No |
| V20.4 | Motorcycle Driver Injured In Collision With Pedestrian Or Animal In Traffic Accident | Yes | No |
| V20.5 | Motorcycle Passenger Injured In Collision With Pedestrian Or Animal In Traffic Accident | Yes | No |
| V20.9 | Unspecified Motorcycle Rider Injured In Collision With Pedestrian Or Animal In Traffic Accident | Yes | No |
| V21.0 | Motorcycle Driver Injured In Collision With Pedal Cycle In Nontraffic Accident | Yes | No |
| V21.1 | Motorcycle Passenger Injured In Collision With Pedal Cycle In Nontraffic Accident | Yes | No |
| V21.2 | Unspecified Motorcycle Rider Injured In Collision With Pedal Cycle In Nontraffic Accident | Yes | No |
| V21.3 | Person Boarding Or Alighting A Motorcycle Injured In Collision With Pedal Cycle | Yes | No |
| V21.4 | Motorcycle Driver Injured In Collision With Pedal Cycle In Traffic Accident | Yes | No |
| V21.5 | Motorcycle Passenger Injured In Collision With Pedal Cycle In Traffic Accident | Yes | No |
| V21.9 | Unspecified Motorcycle Rider Injured In Collision With Pedal Cycle In Traffic Accident | Yes | No |
| V22.0 | Motorcycle Driver Injured In Collision With Two- Or Three-Wheeled Motor Vehicle In Nontraffic Accident | Yes | No |
| V22.1 | Motorcycle Passenger Injured In Collision With Two- Or Three-Wheeled Motor Vehicle In Nontraffic Accident | Yes | No |
| V22.2 | Unspecified Motorcycle Rider Injured In Collision With Two- Or Three-Wheeled Motor Vehicle In Nontraffic Accident | Yes | No |
| V22.3 | Person Boarding Or Alighting A Motorcycle Injured In Collision With Two- Or Three-Wheeled Motor Vehicle | Yes | No |
| V22.4 | Motorcycle Driver Injured In Collision With Two- Or Three-Wheeled Motor Vehicle In Traffic Accident | Yes | No |
| V22.5 | Motorcycle Passenger Injured In Collision With Two- Or Three-Wheeled Motor Vehicle In Traffic Accident | Yes | No |
| V22.9 | Unspecified Motorcycle Rider Injured In Collision With Two- Or Three-Wheeled Motor Vehicle In Traffic Accident | Yes | No |
| V23.0 | Motorcycle Driver Injured In Collision With Car, Pick-Up Truck Or Van In Nontraffic Accident | Yes | No |
| V23.1 | Motorcycle Passenger Injured In Collision With Car, Pick-Up Truck Or Van In Nontraffic Accident | Yes | No |
| V23.2 | Unspecified Motorcycle Rider Injured In Collision With Car, Pick-Up Truck Or Van In Nontraffic Accident | Yes | No |
| V23.3 | Person Boarding Or Alighting A Motorcycle Injured In Collision With Car, Pick-Up Truck Or Van | Yes | No |
| V23.4 | Motorcycle Driver Injured In Collision With Car, Pick-Up Truck Or Van In Traffic Accident | Yes | No |
| V23.5 | Motorcycle Passenger Injured In Collision With Car, Pick-Up Truck Or Van In Traffic Accident | Yes | No |
| V23.9 | Unspecified Motorcycle Rider Injured In Collision With Car, Pick-Up Truck Or Van In Traffic Accident | Yes | No |
| V24.0 | Motorcycle Driver Injured In Collision With Heavy Transport Vehicle Or Bus In Nontraffic Accident | Yes | No |
| V24.1 | Motorcycle Passenger Injured In Collision With Heavy Transport Vehicle Or Bus In Nontraffic Accident | Yes | No |
| V24.2 | Unspecified Motorcycle Rider Injured In Collision With Heavy Transport Vehicle Or Bus In Nontraffic Accident | Yes | No |
| V24.3 | Person Boarding Or Alighting A Motorcycle Injured In Collision With Heavy Transport Vehicle Or Bus | Yes | No |
| V24.4 | Motorcycle Driver Injured In Collision With Heavy Transport Vehicle Or Bus In Traffic Accident | Yes | No |
| V24.5 | Motorcycle Passenger Injured In Collision With Heavy Transport Vehicle Or Bus In Traffic Accident | Yes | No |
| V24.9 | Unspecified Motorcycle Rider Injured In Collision With Heavy Transport Vehicle Or Bus In Traffic Accident | Yes | No |
| V25.0 | Motorcycle Driver Injured In Collision With Railway Train Or Railway Vehicle In Nontraffic Accident | Yes | No |
| V25.1 | Motorcycle Passenger Injured In Collision With Railway Train Or Railway Vehicle In Nontraffic Accident | Yes | No |
| V25.2 | Unspecified Motorcycle Rider Injured In Collision With Railway Train Or Railway Vehicle In Nontraffic Accident | Yes | No |
| V25.3 | Person Boarding Or Alighting A Motorcycle Injured In Collision With Railway Train Or Railway Vehicle | Yes | No |
| V25.4 | Motorcycle Driver Injured In Collision With Railway Train Or Railway Vehicle In Traffic Accident | Yes | No |
| V25.5 | Motorcycle Passenger Injured In Collision With Railway Train Or Railway Vehicle In Traffic Accident | Yes | No |
| V25.9 | Unspecified Motorcycle Rider Injured In Collision With Railway Train Or Railway Vehicle In Traffic Accident | Yes | No |
| V26.0 | Motorcycle Driver Injured In Collision With Other Nonmotor Vehicle In Nontraffic Accident | Yes | No |
| V26.1 | Motorcycle Passenger Injured In Collision With Other Nonmotor Vehicle In Nontraffic Accident | Yes | No |
| V26.2 | Unspecified Motorcycle Rider Injured In Collision With Other Nonmotor Vehicle In Nontraffic Accident | Yes | No |
| V26.3 | Person Boarding Or Alighting A Motorcycle Injured In Collision With Other Nonmotor Vehicle | Yes | No |
| V26.4 | Motorcycle Driver Injured In Collision With Other Nonmotor Vehicle In Traffic Accident | Yes | No |
| V26.5 | Motorcycle Passenger Injured In Collision With Other Nonmotor Vehicle In Traffic Accident | Yes | No |
| V26.9 | Unspecified Motorcycle Rider Injured In Collision With Other Nonmotor Vehicle In Traffic Accident | Yes | No |
| V27.0 | Motorcycle Driver Injured In Collision With Fixed Or Stationary Object In Nontraffic Accident | Yes | No |
| V27.1 | Motorcycle Passenger Injured In Collision With Fixed Or Stationary Object In Nontraffic Accident | Yes | No |
| V27.2 | Unspecified Motorcycle Rider Injured In Collision With Fixed Or Stationary Object In Nontraffic Accident | Yes | No |
| V27.3 | Person Boarding Or Alighting A Motorcycle Injured In Collision With Fixed Or Stationary Object | Yes | No |
| V27.4 | Motorcycle Driver Injured In Collision With Fixed Or Stationary Object In Traffic Accident | Yes | No |
| V27.5 | Motorcycle Passenger Injured In Collision With Fixed Or Stationary Object In Traffic Accident | Yes | No |
| V27.9 | Unspecified Motorcycle Rider Injured In Collision With Fixed Or Stationary Object In Traffic Accident | Yes | No |
| V28.0 | Motorcycle Driver Injured In Noncollision Transport Accident In Nontraffic Accident | Yes | No |
| V28.1 | Motorcycle Passenger Injured In Noncollision Transport Accident In Nontraffic Accident | Yes | No |
| V28.2 | Unspecified Motorcycle Rider Injured In Noncollision Transport Accident In Nontraffic Accident | Yes | No |
| V28.3 | Person Boarding Or Alighting A Motorcycle Injured In Noncollision Transport Accident | Yes | No |
| V28.4 | Motorcycle Driver Injured In Noncollision Transport Accident In Traffic Accident | Yes | No |
| V28.5 | Motorcycle Passenger Injured In Noncollision Transport Accident In Traffic Accident | Yes | No |
| V28.9 | Unspecified Motorcycle Rider Injured In Noncollision Transport Accident In Traffic Accident | Yes | No |
| V29.0 | Motorcycle Driver Injured In Collision With Other And Unspecified Motor Vehicles In Nontraffic Accident | Yes | No |
| V29.1 | Motorcycle Passenger Injured In Collision With Other And Unspecified Motor Vehicles In Nontraffic Accident | Yes | No |
| V29.2 | Unspecified Motorcycle Rider Injured In Collision With Other And Unspecified Motor Vehicles In Nontraffic Accident | Yes | No |
| V29.3 | Motorcycle Rider (Driver) (Passenger) Injured In Unspecified Nontraffic Accident | Yes | No |
| V29.4 | Motorcycle Driver Injured In Collision With Other And Unspecified Motor Vehicles In Traffic Accident | Yes | No |
| V29.5 | Motorcycle Passenger Injured In Collision With Other And Unspecified Motor Vehicles In Traffic Accident | Yes | No |
| V29.6 | Unspecified Motorcycle Rider Injured In Collision With Other And Unspecified Motor Vehicles In Traffic Accident | Yes | No |
| V29.8 | Motorcycle Rider (Driver) (Passenger) Injured In Other Specified Transport Accidents | Yes | No |
| V29.9 | Motorcycle Rider (Driver) (Passenger) Injured In Unspecified Traffic Accident | Yes | No |
| V30.0 | Driver Of Three-Wheeled Motor Vehicle Injured In Collision With Pedestrian Or Animal In Nontraffic Accident | Yes | No |
| V30.1 | Passenger In Three-Wheeled Motor Vehicle Injured In Collision With Pedestrian Or Animal In Nontraffic Accident | Yes | No |
| V30.2 | Person On Outside Of Three-Wheeled Motor Vehicle Injured In Collision With Pedestrian Or Animal In Nontraffic Accident | Yes | No |
| V30.3 | Unspecified Occupant Of Three-Wheeled Motor Vehicle Injured In Collision With Pedestrian Or Animal In Nontraffic Accident | Yes | No |
| V30.4 | Person Boarding Or Alighting A Three-Wheeled Motor Vehicle Injured In Collision With Pedestrian Or Animal | Yes | No |
| V30.5 | Driver Of Three-Wheeled Motor Vehicle Injured In Collision With Pedestrian Or Animal In Traffic Accident | Yes | No |
| V30.6 | Passenger In Three-Wheeled Motor Vehicle Injured In Collision With Pedestrian Or Animal In Traffic Accident | Yes | No |
| V30.7 | Person On Outside Of Three-Wheeled Motor Vehicle Injured In Collision With Pedestrian Or Animal In Traffic Accident | Yes | No |
| V30.9 | Unspecified Occupant Of Three-Wheeled Motor Vehicle Injured In Collision With Pedestrian Or Animal In Traffic Accident | Yes | No |
| V31.0 | Driver Of Three-Wheeled Motor Vehicle Injured In Collision With Pedal Cycle In Nontraffic Accident | Yes | No |
| V31.1 | Passenger In Three-Wheeled Motor Vehicle Injured In Collision With Pedal Cycle In Nontraffic Accident | Yes | No |
| V31.2 | Person On Outside Of Three-Wheeled Motor Vehicle Injured In Collision With Pedal Cycle In Nontraffic Accident | Yes | No |
| V31.3 | Unspecified Occupant Of Three-Wheeled Motor Vehicle Injured In Collision With Pedal Cycle In Nontraffic Accident | Yes | No |
| V31.4 | Person Boarding Or Alighting A Three-Wheeled Motor Vehicle Injured In Collision With Pedal Cycle | Yes | No |
| V31.5 | Driver Of Three-Wheeled Motor Vehicle Injured In Collision With Pedal Cycle In Traffic Accident | Yes | No |
| V31.6 | Passenger In Three-Wheeled Motor Vehicle Injured In Collision With Pedal Cycle In Traffic Accident | Yes | No |
| V31.7 | Person On Outside Of Three-Wheeled Motor Vehicle Injured In Collision With Pedal Cycle In Traffic Accident | Yes | No |
| V31.9 | Unspecified Occupant Of Three-Wheeled Motor Vehicle Injured In Collision With Pedal Cycle In Traffic Accident | Yes | No |
| V32.0 | Driver Of Three-Wheeled Motor Vehicle Injured In Collision With Two- Or Three-Wheeled Motor Vehicle In Nontraffic Accident | Yes | No |
| V32.1 | Passenger In Three-Wheeled Motor Vehicle Injured In Collision With Two- Or Three-Wheeled Motor Vehicle In Nontraffic Accident | Yes | No |
| V32.2 | Person On Outside Of Three-Wheeled Motor Vehicle Injured In Collision With Two- Or Three-Wheeled Motor Vehicle In Nontraffic Accident | Yes | No |
| V32.3 | Unspecified Occupant Of Three-Wheeled Motor Vehicle Injured In Collision With Two- Or Three-Wheeled Motor Vehicle In Nontraffic Accident | Yes | No |
| V32.4 | Person Boarding Or Alighting A Three-Wheeled Motor Vehicle Injured In Collision With Two- Or Three-Wheeled Motor Vehicle | Yes | No |
| V32.5 | Driver Of Three-Wheeled Motor Vehicle Injured In Collision With Two- Or Three-Wheeled Motor Vehicle In Traffic Accident | Yes | No |
| V32.6 | Passenger In Three-Wheeled Motor Vehicle Injured In Collision With Two- Or Three-Wheeled Motor Vehicle In Traffic Accident | Yes | No |
| V32.7 | Person On Outside Of Three-Wheeled Motor Vehicle Injured In Collision With Two- Or Three-Wheeled Motor Vehicle In Traffic Accident | Yes | No |
| V32.9 | Unspecified Occupant Of Three-Wheeled Motor Vehicle Injured In Collision With Two- Or Three-Wheeled Motor Vehicle In Traffic Accident | Yes | No |
| V33.0 | Driver Of Three-Wheeled Motor Vehicle Injured In Collision With Car, Pick-Up Truck Or Van In Nontraffic Accident | Yes | No |
| V33.1 | Passenger In Three-Wheeled Motor Vehicle Injured In Collision With Car, Pick-Up Truck Or Van In Nontraffic Accident | Yes | No |
| V33.2 | Person On Outside Of Three-Wheeled Motor Vehicle Injured In Collision With Car, Pick-Up Truck Or Van In Nontraffic Accident | Yes | No |
| V33.3 | Unspecified Occupant Of Three-Wheeled Motor Vehicle Injured In Collision With Car, Pick-Up Truck Or Van In Nontraffic Accident | Yes | No |
| V33.4 | Person Boarding Or Alighting A Three-Wheeled Motor Vehicle Injured In Collision With Car, Pick-Up Truck Or Van | Yes | No |
| V33.5 | Driver Of Three-Wheeled Motor Vehicle Injured In Collision With Car, Pick-Up Truck Or Van In Traffic Accident | Yes | No |
| V33.6 | Passenger In Three-Wheeled Motor Vehicle Injured In Collision With Car, Pick-Up Truck Or Van In Traffic Accident | Yes | No |
| V33.7 | Person On Outside Of Three-Wheeled Motor Vehicle Injured In Collision With Car, Pick-Up Truck Or Van In Traffic Accident | Yes | No |
| V33.9 | Unspecified Occupant Of Three-Wheeled Motor Vehicle Injured In Collision With Car, Pick-Up Truck Or Van In Traffic Accident | Yes | No |
| V34.0 | Driver Of Three-Wheeled Motor Vehicle Injured In Collision With Heavy Transport Vehicle Or Bus In Nontraffic Accident | Yes | No |
| V34.1 | Passenger In Three-Wheeled Motor Vehicle Injured In Collision With Heavy Transport Vehicle Or Bus In Nontraffic Accident | Yes | No |
| V34.2 | Person On Outside Of Three-Wheeled Motor Vehicle Injured In Collision With Heavy Transport Vehicle Or Bus In Nontraffic Accident | Yes | No |
| V34.3 | Unspecified Occupant Of Three-Wheeled Motor Vehicle Injured In Collision With Heavy Transport Vehicle Or Bus In Nontraffic Accident | Yes | No |
| V34.4 | Person Boarding Or Alighting A Three-Wheeled Motor Vehicle Injured In Collision With Heavy Transport Vehicle Or Bus | Yes | No |
| V34.5 | Driver Of Three-Wheeled Motor Vehicle Injured In Collision With Heavy Transport Vehicle Or Bus In Traffic Accident | Yes | No |
| V34.6 | Passenger In Three-Wheeled Motor Vehicle Injured In Collision With Heavy Transport Vehicle Or Bus In Traffic Accident | Yes | No |
| V34.7 | Person On Outside Of Three-Wheeled Motor Vehicle Injured In Collision With Heavy Transport Vehicle Or Bus In Traffic Accident | Yes | No |
| V34.9 | Unspecified Occupant Of Three-Wheeled Motor Vehicle Injured In Collision With Heavy Transport Vehicle Or Bus In Traffic Accident | Yes | No |
| V35.0 | Driver Of Three-Wheeled Motor Vehicle Injured In Collision With Railway Train Or Railway Vehicle In Nontraffic Accident | Yes | No |
| V35.1 | Passenger In Three-Wheeled Motor Vehicle Injured In Collision With Railway Train Or Railway Vehicle In Nontraffic Accident | Yes | No |
| V35.2 | Person On Outside Of Three-Wheeled Motor Vehicle Injured In Collision With Railway Train Or Railway Vehicle In Nontraffic Accident | Yes | No |
| V35.3 | Unspecified Occupant Of Three-Wheeled Motor Vehicle Injured In Collision With Railway Train Or Railway Vehicle In Nontraffic Accident | Yes | No |
| V35.4 | Person Boarding Or Alighting A Three-Wheeled Motor Vehicle Injured In Collision With Railway Train Or Railway Vehicle | Yes | No |
| V35.5 | Driver Of Three-Wheeled Motor Vehicle Injured In Collision With Railway Train Or Railway Vehicle In Traffic Accident | Yes | No |
| V35.6 | Passenger In Three-Wheeled Motor Vehicle Injured In Collision With Railway Train Or Railway Vehicle In Traffic Accident | Yes | No |
| V35.7 | Person On Outside Of Three-Wheeled Motor Vehicle Injured In Collision With Railway Train Or Railway Vehicle In Traffic Accident | Yes | No |
| V35.9 | Unspecified Occupant Of Three-Wheeled Motor Vehicle Injured In Collision With Railway Train Or Railway Vehicle In Traffic Accident | Yes | No |
| V36.0 | Driver Of Three-Wheeled Motor Vehicle Injured In Collision With Other Nonmotor Vehicle In Nontraffic Accident | Yes | No |
| V36.1 | Passenger In Three-Wheeled Motor Vehicle Injured In Collision With Other Nonmotor Vehicle In Nontraffic Accident | Yes | No |
| V36.2 | Person On Outside Of Three-Wheeled Motor Vehicle Injured In Collision With Other Nonmotor Vehicle In Nontraffic Accident | Yes | No |
| V36.3 | Unspecified Occupant Of Three-Wheeled Motor Vehicle Injured In Collision With Other Nonmotor Vehicle In Nontraffic Accident | Yes | No |
| V36.4 | Person Boarding Or Alighting A Three-Wheeled Motor Vehicle Injured In Collision With Other Nonmotor Vehicle | Yes | No |
| V36.5 | Driver Of Three-Wheeled Motor Vehicle Injured In Collision With Other Nonmotor Vehicle In Traffic Accident | Yes | No |
| V36.6 | Passenger In Three-Wheeled Motor Vehicle Injured In Collision With Other Nonmotor Vehicle In Traffic Accident | Yes | No |
| V36.7 | Person On Outside Of Three-Wheeled Motor Vehicle Injured In Collision With Other Nonmotor Vehicle In Traffic Accident | Yes | No |
| V36.9 | Unspecified Occupant Of Three-Wheeled Motor Vehicle Injured In Collision With Other Nonmotor Vehicle In Traffic Accident | Yes | No |
| V37.0 | Driver Of Three-Wheeled Motor Vehicle Injured In Collision With Fixed Or Stationary Object In Nontraffic Accident | Yes | No |
| V37.1 | Passenger In Three-Wheeled Motor Vehicle Injured In Collision With Fixed Or Stationary Object In Nontraffic Accident | Yes | No |
| V37.2 | Person On Outside Of Three-Wheeled Motor Vehicle Injured In Collision With Fixed Or Stationary Object In Nontraffic Accident | Yes | No |
| V37.3 | Unspecified Occupant Of Three-Wheeled Motor Vehicle Injured In Collision With Fixed Or Stationary Object In Nontraffic Accident | Yes | No |
| V37.4 | Person Boarding Or Alighting A Three-Wheeled Motor Vehicle Injured In Collision With Fixed Or Stationary Object | Yes | No |
| V37.5 | Driver Of Three-Wheeled Motor Vehicle Injured In Collision With Fixed Or Stationary Object In Traffic Accident | Yes | No |
| V37.6 | Passenger In Three-Wheeled Motor Vehicle Injured In Collision With Fixed Or Stationary Object In Traffic Accident | Yes | No |
| V37.7 | Person On Outside Of Three-Wheeled Motor Vehicle Injured In Collision With Fixed Or Stationary Object In Traffic Accident | Yes | No |
| V37.9 | Unspecified Occupant Of Three-Wheeled Motor Vehicle Injured In Collision With Fixed Or Stationary Object In Traffic Accident | Yes | No |
| V38.0 | Driver Of Three-Wheeled Motor Vehicle Injured In Noncollision Transport Accident In Nontraffic Accident | Yes | No |
| V38.1 | Passenger In Three-Wheeled Motor Vehicle Injured In Noncollision Transport Accident In Nontraffic Accident | Yes | No |
| V38.2 | Person On Outside Of Three-Wheeled Motor Vehicle Injured In Noncollision Transport Accident In Nontraffic Accident | Yes | No |
| V38.3 | Unspecified Occupant Of Three-Wheeled Motor Vehicle Injured In Noncollision Transport Accident In Nontraffic Accident | Yes | No |
| V38.4 | Person Boarding Or Alighting A Three-Wheeled Motor Vehicle Injured In Noncollision Transport Accident | Yes | No |
| V38.5 | Driver Of Three-Wheeled Motor Vehicle Injured In Noncollision Transport Accident In Traffic Accident | Yes | No |
| V38.6 | Passenger In Three-Wheeled Motor Vehicle Injured In Noncollision Transport Accident In Traffic Accident | Yes | No |
| V38.7 | Person On Outside Of Three-Wheeled Motor Vehicle Injured In Noncollision Transport Accident In Traffic Accident | Yes | No |
| V38.9 | Unspecified Occupant Of Three-Wheeled Motor Vehicle Injured In Noncollision Transport Accident In Traffic Accident | Yes | No |
| V39.0 | Driver Of Three-Wheeled Motor Vehicle Injured In Collision With Other And Unspecified Motor Vehicles In Nontraffic Accident | Yes | No |
| V39.1 | Passenger In Three-Wheeled Motor Vehicle Injured In Collision With Other And Unspecified Motor Vehicles In Nontraffic Accident | Yes | No |
| V39.2 | Unspecified Occupant Of Three-Wheeled Motor Vehicle Injured In Collision With Other And Unspecified Motor Vehicles In Nontraffic Accident | Yes | No |
| V39.3 | Occupant (Driver) (Passenger) Of Three-Wheeled Motor Vehicle Injured In Unspecified Nontraffic Accident | Yes | No |
| V39.4 | Driver Of Three-Wheeled Motor Vehicle Injured In Collision With Other And Unspecified Motor Vehicles In Traffic Accident | Yes | No |
| V39.5 | Passenger In Three-Wheeled Motor Vehicle Injured In Collision With Other And Unspecified Motor Vehicles In Traffic Accident | Yes | No |
| V39.6 | Unspecified Occupant Of Three-Wheeled Motor Vehicle Injured In Collision With Other And Unspecified Motor Vehicles In Traffic Accident | Yes | No |
| V39.8 | Occupant (Driver) (Passenger) Of Three-Wheeled Motor Vehicle Injured In Other Specified Transport Accidents | Yes | No |
| V39.9 | Occupant (Driver) (Passenger) Of Three-Wheeled Motor Vehicle Injured In Unspecified Traffic Accident | Yes | No |
| V40.0 | Car Driver Injured In Collision With Pedestrian Or Animal In Nontraffic Accident | Yes | No |
| V40.1 | Car Passenger Injured In Collision With Pedestrian Or Animal In Nontraffic Accident | Yes | No |
| V40.2 | Person On Outside Of Car Injured In Collision With Pedestrian Or Animal In Nontraffic Accident | Yes | No |
| V40.3 | Unspecified Car Occupant Injured In Collision With Pedestrian Or Animal In Nontraffic Accident | Yes | No |
| V40.4 | Person Boarding Or Alighting A Car Injured In Collision With Pedestrian Or Animal | Yes | No |
| V40.5 | Car Driver Injured In Collision With Pedestrian Or Animal In Traffic Accident | Yes | No |
| V40.6 | Car Passenger Injured In Collision With Pedestrian Or Animal In Traffic Accident | Yes | No |
| V40.7 | Person On Outside Of Car Injured In Collision With Pedestrian Or Animal In Traffic Accident | Yes | No |
| V40.9 | Unspecified Car Occupant Injured In Collision With Pedestrian Or Animal In Traffic Accident | Yes | No |
| V41.0 | Car Driver Injured In Collision With Pedal Cycle In Nontraffic Accident | Yes | No |
| V41.1 | Car Passenger Injured In Collision With Pedal Cycle In Nontraffic Accident | Yes | No |
| V41.2 | Person On Outside Of Car Injured In Collision With Pedal Cycle In Nontraffic Accident | Yes | No |
| V41.3 | Unspecified Car Occupant Injured In Collision With Pedal Cycle In Nontraffic Accident | Yes | No |
| V41.4 | Person Boarding Or Alighting A Car Injured In Collision With Pedal Cycle | Yes | No |
| V41.5 | Car Driver Injured In Collision With Pedal Cycle In Traffic Accident | Yes | No |
| V41.6 | Car Passenger Injured In Collision With Pedal Cycle In Traffic Accident | Yes | No |
| V41.7 | Person On Outside Of Car Injured In Collision With Pedal Cycle In Traffic Accident | Yes | No |
| V41.9 | Unspecified Car Occupant Injured In Collision With Pedal Cycle In Traffic Accident | Yes | No |
| V42.0 | Car Driver Injured In Collision With Two- Or Three-Wheeled Motor Vehicle In Nontraffic Accident | Yes | No |
| V42.1 | Car Passenger Injured In Collision With Two- Or Three-Wheeled Motor Vehicle In Nontraffic Accident | Yes | No |
| V42.2 | Person On Outside Of Car Injured In Collision With Two- Or Three-Wheeled Motor Vehicle In Nontraffic Accident | Yes | No |
| V42.3 | Unspecified Car Occupant Injured In Collision With Two- Or Three-Wheeled Motor Vehicle In Nontraffic Accident | Yes | No |
| V42.4 | Person Boarding Or Alighting A Car Injured In Collision With Two- Or Three-Wheeled Motor Vehicle | Yes | No |
| V42.5 | Car Driver Injured In Collision With Two- Or Three-Wheeled Motor Vehicle In Traffic Accident | Yes | No |
| V42.6 | Car Passenger Injured In Collision With Two- Or Three-Wheeled Motor Vehicle In Traffic Accident | Yes | No |
| V42.7 | Person On Outside Of Car Injured In Collision With Two- Or Three-Wheeled Motor Vehicle In Traffic Accident | Yes | No |
| V42.9 | Unspecified Car Occupant Injured In Collision With Two- Or Three-Wheeled Motor Vehicle In Traffic Accident | Yes | No |
| V43.0 | Car Driver Injured In Collision With Car, Pick-Up Truck Or Van In Nontraffic Accident | Yes | No |
| V43.1 | Car Passenger Injured In Collision With Car, Pick-Up Truck Or Van In Nontraffic Accident | Yes | No |
| V43.2 | Person On Outside Of Car Injured In Collision With Car, Pick-Up Truck Or Van In Nontraffic Accident | Yes | No |
| V43.3 | Unspecified Car Occupant Injured In Collision With Car, Pick-Up Truck Or Van In Nontraffic Accident | Yes | No |
| V43.4 | Person Boarding Or Alighting A Car Injured In Collision With Car, Pick-Up Truck Or Van | Yes | No |
| V43.5 | Car Driver Injured In Collision With Car, Pick-Up Truck Or Van In Traffic Accident | Yes | No |
| V43.6 | Car Passenger Injured In Collision With Car, Pick-Up Truck Or Van In Traffic Accident | Yes | No |
| V43.7 | Person On Outside Of Car Injured In Collision With Car, Pick-Up Truck Or Van In Traffic Accident | Yes | No |
| V43.9 | Unspecified Car Occupant Injured In Collision With Car, Pick-Up Truck Or Van In Traffic Accident | Yes | No |
| V44.0 | Car Driver Injured In Collision With Heavy Transport Vehicle Or Bus In Nontraffic Accident | Yes | No |
| V44.1 | Car Passenger Injured In Collision With Heavy Transport Vehicle Or Bus In Nontraffic Accident | Yes | No |
| V44.2 | Person On Outside Of Car Injured In Collision With Heavy Transport Vehicle Or Bus In Nontraffic Accident | Yes | No |
| V44.3 | Unspecified Car Occupant Injured In Collision With Heavy Transport Vehicle Or Bus In Nontraffic Accident | Yes | No |
| V44.4 | Person Boarding Or Alighting A Car Injured In Collision With Heavy Transport Vehicle Or Bus | Yes | No |
| V44.5 | Car Driver Injured In Collision With Heavy Transport Vehicle Or Bus In Traffic Accident | Yes | No |
| V44.6 | Car Passenger Injured In Collision With Heavy Transport Vehicle Or Bus In Traffic Accident | Yes | No |
| V44.7 | Person On Outside Of Car Injured In Collision With Heavy Transport Vehicle Or Bus In Traffic Accident | Yes | No |
| V44.9 | Unspecified Car Occupant Injured In Collision With Heavy Transport Vehicle Or Bus In Traffic Accident | Yes | No |
| V45.0 | Car Driver Injured In Collision With Railway Train Or Railway Vehicle In Nontraffic Accident | Yes | No |
| V45.1 | Car Passenger Injured In Collision With Railway Train Or Railway Vehicle In Nontraffic Accident | Yes | No |
| V45.2 | Person On Outside Of Car Injured In Collision With Railway Train Or Railway Vehicle In Nontraffic Accident | Yes | No |
| V45.3 | Unspecified Car Occupant Injured In Collision With Railway Train Or Railway Vehicle In Nontraffic Accident | Yes | No |
| V45.4 | Person Boarding Or Alighting A Car Injured In Collision With Railway Train Or Railway Vehicle | Yes | No |
| V45.5 | Car Driver Injured In Collision With Railway Train Or Railway Vehicle In Traffic Accident | Yes | No |
| V45.6 | Car Passenger Injured In Collision With Railway Train Or Railway Vehicle In Traffic Accident | Yes | No |
| V45.7 | Person On Outside Of Car Injured In Collision With Railway Train Or Railway Vehicle In Traffic Accident | Yes | No |
| V45.9 | Unspecified Car Occupant Injured In Collision With Railway Train Or Railway Vehicle In Traffic Accident | Yes | No |
| V46.0 | Car Driver Injured In Collision With Other Nonmotor Vehicle In Nontraffic Accident | Yes | No |
| V46.1 | Car Passenger Injured In Collision With Other Nonmotor Vehicle In Nontraffic Accident | Yes | No |
| V46.2 | Person On Outside Of Car Injured In Collision With Other Nonmotor Vehicle In Nontraffic Accident | Yes | No |
| V46.3 | Unspecified Car Occupant Injured In Collision With Other Nonmotor Vehicle In Nontraffic Accident | Yes | No |
| V46.4 | Person Boarding Or Alighting A Car Injured In Collision With Other Nonmotor Vehicle | Yes | No |
| V46.5 | Car Driver Injured In Collision With Other Nonmotor Vehicle In Traffic Accident | Yes | No |
| V46.6 | Car Passenger Injured In Collision With Other Nonmotor Vehicle In Traffic Accident | Yes | No |
| V46.7 | Person On Outside Of Car Injured In Collision With Other Nonmotor Vehicle In Traffic Accident | Yes | No |
| V46.9 | Unspecified Car Occupant Injured In Collision With Other Nonmotor Vehicle In Traffic Accident | Yes | No |
| V47.0 | Car Driver Injured In Collision With Fixed Or Stationary Object In Nontraffic Accident | Yes | No |
| V47.1 | Car Passenger Injured In Collision With Fixed Or Stationary Object In Nontraffic Accident | Yes | No |
| V47.2 | Person On Outside Of Car Injured In Collision With Fixed Or Stationary Object In Nontraffic Accident | Yes | No |
| V47.3 | Unspecified Car Occupant Injured In Collision With Fixed Or Stationary Object In Nontraffic Accident | Yes | No |
| V47.4 | Person Boarding Or Alighting A Car Injured In Collision With Fixed Or Stationary Object | Yes | No |
| V47.5 | Car Driver Injured In Collision With Fixed Or Stationary Object In Traffic Accident | Yes | No |
| V47.6 | Car Passenger Injured In Collision With Fixed Or Stationary Object In Traffic Accident | Yes | No |
| V47.7 | Person On Outside Of Car Injured In Collision With Fixed Or Stationary Object In Traffic Accident | Yes | No |
| V47.9 | Unspecified Car Occupant Injured In Collision With Fixed Or Stationary Object In Traffic Accident | Yes | No |
| V48.0 | Car Driver Injured In Noncollision Transport Accident In Nontraffic Accident | Yes | No |
| V48.1 | Car Passenger Injured In Noncollision Transport Accident In Nontraffic Accident | Yes | No |
| V48.2 | Person On Outside Of Car Injured In Noncollision Transport Accident In Nontraffic Accident | Yes | No |
| V48.3 | Unspecified Car Occupant Injured In Noncollision Transport Accident In Nontraffic Accident | Yes | No |
| V48.4 | Person Boarding Or Alighting A Car Injured In Noncollision Transport Accident | Yes | No |
| V48.5 | Car Driver Injured In Noncollision Transport Accident In Traffic Accident | Yes | No |
| V48.6 | Car Passenger Injured In Noncollision Transport Accident In Traffic Accident | Yes | No |
| V48.7 | Person On Outside Of Car Injured In Noncollision Transport Accident In Traffic Accident | Yes | No |
| V48.9 | Unspecified Car Occupant Injured In Noncollision Transport Accident In Traffic Accident | Yes | No |
| V49.0 | Driver Injured In Collision With Other And Unspecified Motor Vehicles In Nontraffic Accident | Yes | No |
| V49.1 | Passenger Injured In Collision With Other And Unspecified Motor Vehicles In Nontraffic Accident | Yes | No |
| V49.2 | Unspecified Car Occupant Injured In Collision With Other And Unspecified Motor Vehicles In Nontraffic Accident | Yes | No |
| V49.3 | Car Occupant (Driver) (Passenger) Injured In Unspecified Nontraffic Accident | Yes | No |
| V49.4 | Driver Injured In Collision With Other And Unspecified Motor Vehicles In Traffic Accident | Yes | No |
| V49.5 | Passenger Injured In Collision With Other And Unspecified Motor Vehicles In Traffic Accident | Yes | No |
| V49.6 | Unspecified Car Occupant Injured In Collision With Other And Unspecified Motor Vehicles In Traffic Accident | Yes | No |
| V49.8 | Car Occupant (Driver) (Passenger) Injured In Other Specified Transport Accidents | Yes | No |
| V49.9 | Car Occupant (Driver) (Passenger) Injured In Unspecified Traffic Accident | Yes | No |
| V50.0 | Driver Of Pick-Up Truck Or Van Injured In Collision With Pedestrian Or Animal In Nontraffic Accident | Yes | No |
| V50.1 | Passenger In Pick-Up Truck Or Van Injured In Collision With Pedestrian Or Animal In Nontraffic Accident | Yes | No |
| V50.2 | Person On Outside Of Pick-Up Truck Or Van Injured In Collision With Pedestrian Or Animal In Nontraffic Accident | Yes | No |
| V50.3 | Unspecified Occupant Of Pick-Up Truck Or Van Injured In Collision With Pedestrian Or Animal In Nontraffic Accident | Yes | No |
| V50.4 | Person Boarding Or Alighting A Pick-Up Truck Or Van Injured In Collision With Pedestrian Or Animal | Yes | No |
| V50.5 | Driver Of Pick-Up Truck Or Van Injured In Collision With Pedestrian Or Animal In Traffic Accident | Yes | No |
| V50.6 | Passenger In Pick-Up Truck Or Van Injured In Collision With Pedestrian Or Animal In Traffic Accident | Yes | No |
| V50.7 | Person On Outside Of Pick-Up Truck Or Van Injured In Collision With Pedestrian Or Animal In Traffic Accident | Yes | No |
| V50.9 | Unspecified Occupant Of Pick-Up Truck Or Van Injured In Collision With Pedestrian Or Animal In Traffic Accident | Yes | No |
| V51.0 | Driver Of Pick-Up Truck Or Van Injured In Collision With Pedal Cycle In Nontraffic Accident | Yes | No |
| V51.1 | Passenger In Pick-Up Truck Or Van Injured In Collision With Pedal Cycle In Nontraffic Accident | Yes | No |
| V51.2 | Person On Outside Of Pick-Up Truck Or Van Injured In Collision With Pedal Cycle In Nontraffic Accident | Yes | No |
| V51.3 | Unspecified Occupant Of Pick-Up Truck Or Van Injured In Collision With Pedal Cycle In Nontraffic Accident | Yes | No |
| V51.4 | Person Boarding Or Alighting A Pick-Up Truck Or Van Injured In Collision With Pedal Cycle | Yes | No |
| V51.5 | Driver Of Pick-Up Truck Or Van Injured In Collision With Pedal Cycle In Traffic Accident | Yes | No |
| V51.6 | Passenger In Pick-Up Truck Or Van Injured In Collision With Pedal Cycle In Traffic Accident | Yes | No |
| V51.7 | Person On Outside Of Pick-Up Truck Or Van Injured In Collision With Pedal Cycle In Traffic Accident | Yes | No |
| V51.9 | Unspecified Occupant Of Pick-Up Truck Or Van Injured In Collision With Pedal Cycle In Traffic Accident | Yes | No |
| V52.0 | Driver Of Pick-Up Truck Or Van Injured In Collision With Two- Or Three-Wheeled Motor Vehicle In Nontraffic Accident | Yes | No |
| V52.1 | Passenger In Pick-Up Truck Or Van Injured In Collision With Two- Or Three-Wheeled Motor Vehicle In Nontraffic Accident | Yes | No |
| V52.2 | Person On Outside Of Pick-Up Truck Or Van Injured In Collision With Two- Or Three-Wheeled Motor Vehicle In Nontraffic Accident | Yes | No |
| V52.3 | Unspecified Occupant Of Pick-Up Truck Or Van Injured In Collision With Two- Or Three-Wheeled Motor Vehicle In Nontraffic Accident | Yes | No |
| V52.4 | Person Boarding Or Alighting A Pick-Up Truck Or Van Injured In Collision With Two- Or Three-Wheeled Motor Vehicle | Yes | No |
| V52.5 | Driver Of Pick-Up Truck Or Van Injured In Collision With Two- Or Three-Wheeled Motor Vehicle In Traffic Accident | Yes | No |
| V52.6 | Passenger In Pick-Up Truck Or Van Injured In Collision With Two- Or Three-Wheeled Motor Vehicle In Traffic Accident | Yes | No |
| V52.7 | Person On Outside Of Pick-Up Truck Or Van Injured In Collision With Two- Or Three-Wheeled Motor Vehicle In Traffic Accident | Yes | No |
| V52.9 | Unspecified Occupant Of Pick-Up Truck Or Van Injured In Collision With Two- Or Three-Wheeled Motor Vehicle In Traffic Accident | Yes | No |
| V53.0 | Driver Of Pick-Up Truck Or Van Injured In Collision With Car, Pick-Up Truck Or Van In Nontraffic Accident | Yes | No |
| V53.1 | Passenger In Pick-Up Truck Or Van Injured In Collision With Car, Pick-Up Truck Or Van In Nontraffic Accident | Yes | No |
| V53.2 | Person On Outside Of Pick-Up Truck Or Van Injured In Collision With Car, Pick-Up Truck Or Van In Nontraffic Accident | Yes | No |
| V53.3 | Unspecified Occupant Of Pick-Up Truck Or Van Injured In Collision With Car, Pick-Up Truck Or Van In Nontraffic Accident | Yes | No |
| V53.4 | Person Boarding Or Alighting A Pick-Up Truck Or Van Injured In Collision With Car, Pick-Up Truck Or Van | Yes | No |
| V53.5 | Driver Of Pick-Up Truck Or Van Injured In Collision With Car, Pick-Up Truck Or Van In Traffic Accident | Yes | No |
| V53.6 | Passenger In Pick-Up Truck Or Van Injured In Collision With Car, Pick-Up Truck Or Van In Traffic Accident | Yes | No |
| V53.7 | Person On Outside Of Pick-Up Truck Or Van Injured In Collision With Car, Pick-Up Truck Or Van In Traffic Accident | Yes | No |
| V53.9 | Unspecified Occupant Of Pick-Up Truck Or Van Injured In Collision With Car, Pick-Up Truck Or Van In Traffic Accident | Yes | No |
| V54.0 | Driver Of Pick-Up Truck Or Van Injured In Collision With Heavy Transport Vehicle Or Bus In Nontraffic Accident | Yes | No |
| V54.1 | Passenger In Pick-Up Truck Or Van Injured In Collision With Heavy Transport Vehicle Or Bus In Nontraffic Accident | Yes | No |
| V54.2 | Person On Outside Of Pick-Up Truck Or Van Injured In Collision With Heavy Transport Vehicle Or Bus In Nontraffic Accident | Yes | No |
| V54.3 | Unspecified Occupant Of Pick-Up Truck Or Van Injured In Collision With Heavy Transport Vehicle Or Bus In Nontraffic Accident | Yes | No |
| V54.4 | Person Boarding Or Alighting A Pick-Up Truck Or Van Injured In Collision With Heavy Transport Vehicle Or Bus | Yes | No |
| V54.5 | Driver Of Pick-Up Truck Or Van Injured In Collision With Heavy Transport Vehicle Or Bus In Traffic Accident | Yes | No |
| V54.6 | Passenger In Pick-Up Truck Or Van Injured In Collision With Heavy Transport Vehicle Or Bus In Traffic Accident | Yes | No |
| V54.7 | Person On Outside Of Pick-Up Truck Or Van Injured In Collision With Heavy Transport Vehicle Or Bus In Traffic Accident | Yes | No |
| V54.9 | Unspecified Occupant Of Pick-Up Truck Or Van Injured In Collision With Heavy Transport Vehicle Or Bus In Traffic Accident | Yes | No |
| V55.0 | Driver Of Pick-Up Truck Or Van Injured In Collision With Railway Train Or Railway Vehicle In Nontraffic Accident | Yes | No |
| V55.1 | Passenger In Pick-Up Truck Or Van Injured In Collision With Railway Train Or Railway Vehicle In Nontraffic Accident | Yes | No |
| V55.2 | Person On Outside Of Pick-Up Truck Or Van Injured In Collision With Railway Train Or Railway Vehicle In Nontraffic Accident | Yes | No |
| V55.3 | Unspecified Occupant Of Pick-Up Truck Or Van Injured In Collision With Railway Train Or Railway Vehicle In Nontraffic Accident | Yes | No |
| V55.4 | Person Boarding Or Alighting A Pick-Up Truck Or Van Injured In Collision With Railway Train Or Railway Vehicle | Yes | No |
| V55.5 | Driver Of Pick-Up Truck Or Van Injured In Collision With Railway Train Or Railway Vehicle In Traffic Accident | Yes | No |
| V55.6 | Passenger In Pick-Up Truck Or Van Injured In Collision With Railway Train Or Railway Vehicle In Traffic Accident | Yes | No |
| V55.7 | Person On Outside Of Pick-Up Truck Or Van Injured In Collision With Railway Train Or Railway Vehicle In Traffic Accident | Yes | No |
| V55.9 | Unspecified Occupant Of Pick-Up Truck Or Van Injured In Collision With Railway Train Or Railway Vehicle In Traffic Accident | Yes | No |
| V56.0 | Driver Of Pick-Up Truck Or Van Injured In Collision With Other Nonmotor Vehicle In Nontraffic Accident | Yes | No |
| V56.1 | Passenger In Pick-Up Truck Or Van Injured In Collision With Other Nonmotor Vehicle In Nontraffic Accident | Yes | No |
| V56.2 | Person On Outside Of Pick-Up Truck Or Van Injured In Collision With Other Nonmotor Vehicle In Nontraffic Accident | Yes | No |
| V56.3 | Unspecified Occupant Of Pick-Up Truck Or Van Injured In Collision With Other Nonmotor Vehicle In Nontraffic Accident | Yes | No |
| V56.4 | Person Boarding Or Alighting A Pick-Up Truck Or Van Injured In Collision With Other Nonmotor Vehicle | Yes | No |
| V56.5 | Driver Of Pick-Up Truck Or Van Injured In Collision With Other Nonmotor Vehicle In Traffic Accident | Yes | No |
| V56.6 | Passenger In Pick-Up Truck Or Van Injured In Collision With Other Nonmotor Vehicle In Traffic Accident | Yes | No |
| V56.7 | Person On Outside Of Pick-Up Truck Or Van Injured In Collision With Other Nonmotor Vehicle In Traffic Accident | Yes | No |
| V56.9 | Unspecified Occupant Of Pick-Up Truck Or Van Injured In Collision With Other Nonmotor Vehicle In Traffic Accident | Yes | No |
| V57.0 | Driver Of Pick-Up Truck Or Van Injured In Collision With Fixed Or Stationary Object In Nontraffic Accident | Yes | No |
| V57.1 | Passenger In Pick-Up Truck Or Van Injured In Collision With Fixed Or Stationary Object In Nontraffic Accident | Yes | No |
| V57.2 | Person On Outside Of Pick-Up Truck Or Van Injured In Collision With Fixed Or Stationary Object In Nontraffic Accident | Yes | No |
| V57.3 | Unspecified Occupant Of Pick-Up Truck Or Van Injured In Collision With Fixed Or Stationary Object In Nontraffic Accident | Yes | No |
| V57.4 | Person Boarding Or Alighting A Pick-Up Truck Or Van Injured In Collision With Fixed Or Stationary Object | Yes | No |
| V57.5 | Driver Of Pick-Up Truck Or Van Injured In Collision With Fixed Or Stationary Object In Traffic Accident | Yes | No |
| V57.6 | Passenger In Pick-Up Truck Or Van Injured In Collision With Fixed Or Stationary Object In Traffic Accident | Yes | No |
| V57.7 | Person On Outside Of Pick-Up Truck Or Van Injured In Collision With Fixed Or Stationary Object In Traffic Accident | Yes | No |
| V57.9 | Unspecified Occupant Of Pick-Up Truck Or Van Injured In Collision With Fixed Or Stationary Object In Traffic Accident | Yes | No |
| V58.0 | Driver Of Pick-Up Truck Or Van Injured In Noncollision Transport Accident In Nontraffic Accident | Yes | No |
| V58.1 | Passenger In Pick-Up Truck Or Van Injured In Noncollision Transport Accident In Nontraffic Accident | Yes | No |
| V58.2 | Person On Outside Of Pick-Up Truck Or Van Injured In Noncollision Transport Accident In Nontraffic Accident | Yes | No |
| V58.3 | Unspecified Occupant Of Pick-Up Truck Or Van Injured In Noncollision Transport Accident In Nontraffic Accident | Yes | No |
| V58.4 | Person Boarding Or Alighting A Pick-Up Truck Or Van Injured In Noncollision Transport Accident | Yes | No |
| V58.5 | Driver Of Pick-Up Truck Or Van Injured In Noncollision Transport Accident In Traffic Accident | Yes | No |
| V58.6 | Passenger In Pick-Up Truck Or Van Injured In Noncollision Transport Accident In Traffic Accident | Yes | No |
| V58.7 | Person On Outside Of Pick-Up Truck Or Van Injured In Noncollision Transport Accident In Traffic Accident | Yes | No |
| V58.9 | Unspecified Occupant Of Pick-Up Truck Or Van Injured In Noncollision Transport Accident In Traffic Accident | Yes | No |
| V59.0 | Driver Of Pick-Up Truck Or Van Injured In Collision With Other And Unspecified Motor Vehicles In Nontraffic Accident | Yes | No |
| V59.1 | Passenger In Pick-Up Truck Or Van Injured In Collision With Other And Unspecified Motor Vehicles In Nontraffic Accident | Yes | No |
| V59.2 | Unspecified Occupant Of Pick-Up Truck Or Van Injured In Collision With Other And Unspecified Motor Vehicles In Nontraffic Accident | Yes | No |
| V59.3 | Occupant (Driver) (Passenger) Of Pick-Up Truck Or Van Injured In Unspecified Nontraffic Accident | Yes | No |
| V59.4 | Driver Of Pick-Up Truck Or Van Injured In Collision With Other And Unspecified Motor Vehicles In Traffic Accident | Yes | No |
| V59.5 | Passenger In Pick-Up Truck Or Van Injured In Collision With Other And Unspecified Motor Vehicles In Traffic Accident | Yes | No |
| V59.6 | Unspecified Occupant Of Pick-Up Truck Or Van Injured In Collision With Other And Unspecified Motor Vehicles In Traffic Accident | Yes | No |
| V59.8 | Occupant (Driver) (Passenger) Of Pick-Up Truck Or Van Injured In Other Specified Transport Accidents | Yes | No |
| V59.9 | Occupant (Driver) (Passenger) Of Pick-Up Truck Or Van Injured In Unspecified Traffic Accident | Yes | No |
| V60.0 | Driver Of Heavy Transport Vehicle Injured In Collision With Pedestrian Or Animal In Nontraffic Accident | Yes | No |
| V60.1 | Passenger In Heavy Transport Vehicle Injured In Collision With Pedestrian Or Animal In Nontraffic Accident | Yes | No |
| V60.2 | Person On Outside Of Heavy Transport Vehicle Injured In Collision With Pedestrian Or Animal In Nontraffic Accident | Yes | No |
| V60.3 | Unspecified Occupant Of Heavy Transport Vehicle Injured In Collision With Pedestrian Or Animal In Nontraffic Accident | Yes | No |
| V60.4 | Person Boarding Or Alighting A Heavy Transport Vehicle Injured In Collision With Pedestrian Or Animal | Yes | No |
| V60.5 | Driver Of Heavy Transport Vehicle Injured In Collision With Pedestrian Or Animal In Traffic Accident | Yes | No |
| V60.6 | Passenger In Heavy Transport Vehicle Injured In Collision With Pedestrian Or Animal In Traffic Accident | Yes | No |
| V60.7 | Person On Outside Of Heavy Transport Vehicle Injured In Collision With Pedestrian Or Animal In Traffic Accident | Yes | No |
| V60.9 | Unspecified Occupant Of Heavy Transport Vehicle Injured In Collision With Pedestrian Or Animal In Traffic Accident | Yes | No |
| V61.0 | Driver Of Heavy Transport Vehicle Injured In Collision With Pedal Cycle In Nontraffic Accident | Yes | No |
| V61.1 | Passenger In Heavy Transport Vehicle Injured In Collision With Pedal Cycle In Nontraffic Accident | Yes | No |
| V61.2 | Person On Outside Of Heavy Transport Vehicle Injured In Collision With Pedal Cycle In Nontraffic Accident | Yes | No |
| V61.3 | Unspecified Occupant Of Heavy Transport Vehicle Injured In Collision With Pedal Cycle In Nontraffic Accident | Yes | No |
| V61.4 | Person Boarding Or Alighting A Heavy Transport Vehicle Injured In Collision With Pedal Cycle While Boarding Or Alighting | Yes | No |
| V61.5 | Driver Of Heavy Transport Vehicle Injured In Collision With Pedal Cycle In Traffic Accident | Yes | No |
| V61.6 | Passenger In Heavy Transport Vehicle Injured In Collision With Pedal Cycle In Traffic Accident | Yes | No |
| V61.7 | Person On Outside Of Heavy Transport Vehicle Injured In Collision With Pedal Cycle In Traffic Accident | Yes | No |
| V61.9 | Unspecified Occupant Of Heavy Transport Vehicle Injured In Collision With Pedal Cycle In Traffic Accident | Yes | No |
| V62.0 | Driver Of Heavy Transport Vehicle Injured In Collision With Two- Or Three-Wheeled Motor Vehicle In Nontraffic Accident | Yes | No |
| V62.1 | Passenger In Heavy Transport Vehicle Injured In Collision With Two- Or Three-Wheeled Motor Vehicle In Nontraffic Accident | Yes | No |
| V62.2 | Person On Outside Of Heavy Transport Vehicle Injured In Collision With Two- Or Three-Wheeled Motor Vehicle In Nontraffic Accident | Yes | No |
| V62.3 | Unspecified Occupant Of Heavy Transport Vehicle Injured In Collision With Two- Or Three-Wheeled Motor Vehicle In Nontraffic Accident | Yes | No |
| V62.4 | Person Boarding Or Alighting A Heavy Transport Vehicle Injured In Collision With Two- Or Three-Wheeled Motor Vehicle | Yes | No |
| V62.5 | Driver Of Heavy Transport Vehicle Injured In Collision With Two- Or Three-Wheeled Motor Vehicle In Traffic Accident | Yes | No |
| V62.6 | Passenger In Heavy Transport Vehicle Injured In Collision With Two- Or Three-Wheeled Motor Vehicle In Traffic Accident | Yes | No |
| V62.7 | Person On Outside Of Heavy Transport Vehicle Injured In Collision With Two- Or Three-Wheeled Motor Vehicle In Traffic Accident | Yes | No |
| V62.9 | Unspecified Occupant Of Heavy Transport Vehicle Injured In Collision With Two- Or Three-Wheeled Motor Vehicle In Traffic Accident | Yes | No |
| V63.0 | Driver Of Heavy Transport Vehicle Injured In Collision With Car, Pick-Up Truck Or Van In Nontraffic Accident | Yes | No |
| V63.1 | Passenger In Heavy Transport Vehicle Injured In Collision With Car, Pick-Up Truck Or Van In Nontraffic Accident | Yes | No |
| V63.2 | Person On Outside Of Heavy Transport Vehicle Injured In Collision With Car, Pick-Up Truck Or Van In Nontraffic Accident | Yes | No |
| V63.3 | Unspecified Occupant Of Heavy Transport Vehicle Injured In Collision With Car, Pick-Up Truck Or Van In Nontraffic Accident | Yes | No |
| V63.4 | Person Boarding Or Alighting A Heavy Transport Vehicle Injured In Collision With Car, Pick-Up Truck Or Van | Yes | No |
| V63.5 | Driver Of Heavy Transport Vehicle Injured In Collision With Car, Pick-Up Truck Or Van In Traffic Accident | Yes | No |
| V63.6 | Passenger In Heavy Transport Vehicle Injured In Collision With Car, Pick-Up Truck Or Van In Traffic Accident | Yes | No |
| V63.7 | Person On Outside Of Heavy Transport Vehicle Injured In Collision With Car, Pick-Up Truck Or Van In Traffic Accident | Yes | No |
| V63.9 | Unspecified Occupant Of Heavy Transport Vehicle Injured In Collision With Car, Pick-Up Truck Or Van In Traffic Accident | Yes | No |
| V64.0 | Driver Of Heavy Transport Vehicle Injured In Collision With Heavy Transport Vehicle Or Bus In Nontraffic Accident | Yes | No |
| V64.1 | Passenger In Heavy Transport Vehicle Injured In Collision With Heavy Transport Vehicle Or Bus In Nontraffic Accident | Yes | No |
| V64.2 | Person On Outside Of Heavy Transport Vehicle Injured In Collision With Heavy Transport Vehicle Or Bus In Nontraffic Accident | Yes | No |
| V64.3 | Unspecified Occupant Of Heavy Transport Vehicle Injured In Collision With Heavy Transport Vehicle Or Bus In Nontraffic Accident | Yes | No |
| V64.4 | Person Boarding Or Alighting A Heavy Transport Vehicle Injured In Collision With Heavy Transport Vehicle Or Bus While Boarding Or Alighting | Yes | No |
| V64.5 | Driver Of Heavy Transport Vehicle Injured In Collision With Heavy Transport Vehicle Or Bus In Traffic Accident | Yes | No |
| V64.6 | Passenger In Heavy Transport Vehicle Injured In Collision With Heavy Transport Vehicle Or Bus In Traffic Accident | Yes | No |
| V64.7 | Person On Outside Of Heavy Transport Vehicle Injured In Collision With Heavy Transport Vehicle Or Bus In Traffic Accident | Yes | No |
| V64.9 | Unspecified Occupant Of Heavy Transport Vehicle Injured In Collision With Heavy Transport Vehicle Or Bus In Traffic Accident | Yes | No |
| V65.0 | Driver Of Heavy Transport Vehicle Injured In Collision With Railway Train Or Railway Vehicle In Nontraffic Accident | Yes | No |
| V65.1 | Passenger In Heavy Transport Vehicle Injured In Collision With Railway Train Or Railway Vehicle In Nontraffic Accident | Yes | No |
| V65.2 | Person On Outside Of Heavy Transport Vehicle Injured In Collision With Railway Train Or Railway Vehicle In Nontraffic Accident | Yes | No |
| V65.3 | Unspecified Occupant Of Heavy Transport Vehicle Injured In Collision With Railway Train Or Railway Vehicle In Nontraffic Accident | Yes | No |
| V65.4 | Person Boarding Or Alighting A Heavy Transport Vehicle Injured In Collision With Railway Train Or Railway Vehicle | Yes | No |
| V65.5 | Driver Of Heavy Transport Vehicle Injured In Collision With Railway Train Or Railway Vehicle In Traffic Accident | Yes | No |
| V65.6 | Passenger In Heavy Transport Vehicle Injured In Collision With Railway Train Or Railway Vehicle In Traffic Accident | Yes | No |
| V65.7 | Person On Outside Of Heavy Transport Vehicle Injured In Collision With Railway Train Or Railway Vehicle In Traffic Accident | Yes | No |
| V65.9 | Unspecified Occupant Of Heavy Transport Vehicle Injured In Collision With Railway Train Or Railway Vehicle In Traffic Accident | Yes | No |
| V66.0 | Driver Of Heavy Transport Vehicle Injured In Collision With Other Nonmotor Vehicle In Nontraffic Accident | Yes | No |
| V66.1 | Passenger In Heavy Transport Vehicle Injured In Collision With Other Nonmotor Vehicle In Nontraffic Accident | Yes | No |
| V66.2 | Person On Outside Of Heavy Transport Vehicle Injured In Collision With Other Nonmotor Vehicle In Nontraffic Accident | Yes | No |
| V66.3 | Unspecified Occupant Of Heavy Transport Vehicle Injured In Collision With Other Nonmotor Vehicle In Nontraffic Accident | Yes | No |
| V66.4 | Person Boarding Or Alighting A Heavy Transport Vehicle Injured In Collision With Other Nonmotor Vehicle | Yes | No |
| V66.5 | Driver Of Heavy Transport Vehicle Injured In Collision With Other Nonmotor Vehicle In Traffic Accident | Yes | No |
| V66.6 | Passenger In Heavy Transport Vehicle Injured In Collision With Other Nonmotor Vehicle In Traffic Accident | Yes | No |
| V66.7 | Person On Outside Of Heavy Transport Vehicle Injured In Collision With Other Nonmotor Vehicle In Traffic Accident | Yes | No |
| V66.9 | Unspecified Occupant Of Heavy Transport Vehicle Injured In Collision With Other Nonmotor Vehicle In Traffic Accident | Yes | No |
| V67.0 | Driver Of Heavy Transport Vehicle Injured In Collision With Fixed Or Stationary Object In Nontraffic Accident | Yes | No |
| V67.1 | Passenger In Heavy Transport Vehicle Injured In Collision With Fixed Or Stationary Object In Nontraffic Accident | Yes | No |
| V67.2 | Person On Outside Of Heavy Transport Vehicle Injured In Collision With Fixed Or Stationary Object In Nontraffic Accident | Yes | No |
| V67.3 | Unspecified Occupant Of Heavy Transport Vehicle Injured In Collision With Fixed Or Stationary Object In Nontraffic Accident | Yes | No |
| V67.4 | Person Boarding Or Alighting A Heavy Transport Vehicle Injured In Collision With Fixed Or Stationary Object | Yes | No |
| V67.5 | Driver Of Heavy Transport Vehicle Injured In Collision With Fixed Or Stationary Object In Traffic Accident | Yes | No |
| V67.6 | Passenger In Heavy Transport Vehicle Injured In Collision With Fixed Or Stationary Object In Traffic Accident | Yes | No |
| V67.7 | Person On Outside Of Heavy Transport Vehicle Injured In Collision With Fixed Or Stationary Object In Traffic Accident | Yes | No |
| V67.9 | Unspecified Occupant Of Heavy Transport Vehicle Injured In Collision With Fixed Or Stationary Object In Traffic Accident | Yes | No |
| V68.0 | Driver Of Heavy Transport Vehicle Injured In Noncollision Transport Accident In Nontraffic Accident | Yes | No |
| V68.1 | Passenger In Heavy Transport Vehicle Injured In Noncollision Transport Accident In Nontraffic Accident | Yes | No |
| V68.2 | Person On Outside Of Heavy Transport Vehicle Injured In Noncollision Transport Accident In Nontraffic Accident | Yes | No |
| V68.3 | Unspecified Occupant Of Heavy Transport Vehicle Injured In Noncollision Transport Accident In Nontraffic Accident | Yes | No |
| V68.4 | Person Boarding Or Alighting A Heavy Transport Vehicle Injured In Noncollision Transport Accident | Yes | No |
| V68.5 | Driver Of Heavy Transport Vehicle Injured In Noncollision Transport Accident In Traffic Accident | Yes | No |
| V68.6 | Passenger In Heavy Transport Vehicle Injured In Noncollision Transport Accident In Traffic Accident | Yes | No |
| V68.7 | Person On Outside Of Heavy Transport Vehicle Injured In Noncollision Transport Accident In Traffic Accident | Yes | No |
| V68.9 | Unspecified Occupant Of Heavy Transport Vehicle Injured In Noncollision Transport Accident In Traffic Accident | Yes | No |
| V69.0 | Driver Of Heavy Transport Vehicle Injured In Collision With Other And Unspecified Motor Vehicles In Nontraffic Accident | Yes | No |
| V69.1 | Passenger In Heavy Transport Vehicle Injured In Collision With Other And Unspecified Motor Vehicles In Nontraffic Accident | Yes | No |
| V69.2 | Unspecified Occupant Of Heavy Transport Vehicle Injured In Collision With Other And Unspecified Motor Vehicles In Nontraffic Accident | Yes | No |
| V69.3 | Occupant (Driver) (Passenger) Of Heavy Transport Vehicle Injured In Unspecified Nontraffic Accident | Yes | No |
| V69.4 | Driver Of Heavy Transport Vehicle Injured In Collision With Other And Unspecified Motor Vehicles In Traffic Accident | Yes | No |
| V69.5 | Passenger In Heavy Transport Vehicle Injured In Collision With Other And Unspecified Motor Vehicles In Traffic Accident | Yes | No |
| V69.6 | Unspecified Occupant Of Heavy Transport Vehicle Injured In Collision With Other And Unspecified Motor Vehicles In Traffic Accident | Yes | No |
| V69.8 | Occupant (Driver) (Passenger) Of Heavy Transport Vehicle Injured In Other Specified Transport Accidents | Yes | No |
| V69.9 | Occupant (Driver) (Passenger) Of Heavy Transport Vehicle Injured In Unspecified Traffic Accident | Yes | No |
| V70.0 | Driver Of Bus Injured In Collision With Pedestrian Or Animal In Nontraffic Accident | Yes | No |
| V70.1 | Passenger On Bus Injured In Collision With Pedestrian Or Animal In Nontraffic Accident | Yes | No |
| V70.2 | Person On Outside Of Bus Injured In Collision With Pedestrian Or Animal In Nontraffic Accident | Yes | No |
| V70.3 | Unspecified Occupant Of Bus Injured In Collision With Pedestrian Or Animal In Nontraffic Accident | Yes | No |
| V70.4 | Person Boarding Or Alighting From Bus Injured In Collision With Pedestrian Or Animal | Yes | No |
| V70.5 | Driver Of Bus Injured In Collision With Pedestrian Or Animal In Traffic Accident | Yes | No |
| V70.6 | Passenger On Bus Injured In Collision With Pedestrian Or Animal In Traffic Accident | Yes | No |
| V70.7 | Person On Outside Of Bus Injured In Collision With Pedestrian Or Animal In Traffic Accident | Yes | No |
| V70.9 | Unspecified Occupant Of Bus Injured In Collision With Pedestrian Or Animal In Traffic Accident | Yes | No |
| V71.0 | Driver Of Bus Injured In Collision With Pedal Cycle In Nontraffic Accident | Yes | No |
| V71.1 | Passenger On Bus Injured In Collision With Pedal Cycle In Nontraffic Accident | Yes | No |
| V71.2 | Person On Outside Of Bus Injured In Collision With Pedal Cycle In Nontraffic Accident | Yes | No |
| V71.3 | Unspecified Occupant Of Bus Injured In Collision With Pedal Cycle In Nontraffic Accident | Yes | No |
| V71.4 | Person Boarding Or Alighting From Bus Injured In Collision With Pedal Cycle | Yes | No |
| V71.5 | Driver Of Bus Injured In Collision With Pedal Cycle In Traffic Accident | Yes | No |
| V71.6 | Passenger On Bus Injured In Collision With Pedal Cycle In Traffic Accident | Yes | No |
| V71.7 | Person On Outside Of Bus Injured In Collision With Pedal Cycle In Traffic Accident | Yes | No |
| V71.9 | Unspecified Occupant Of Bus Injured In Collision With Pedal Cycle In Traffic Accident | Yes | No |
| V72.0 | Driver Of Bus Injured In Collision With Two- Or Three-Wheeled Motor Vehicle In Nontraffic Accident | Yes | No |
| V72.1 | Passenger On Bus Injured In Collision With Two- Or Three-Wheeled Motor Vehicle In Nontraffic Accident | Yes | No |
| V72.2 | Person On Outside Of Bus Injured In Collision With Two- Or Three-Wheeled Motor Vehicle In Nontraffic Accident | Yes | No |
| V72.3 | Unspecified Occupant Of Bus Injured In Collision With Two- Or Three-Wheeled Motor Vehicle In Nontraffic Accident | Yes | No |
| V72.4 | Person Boarding Or Alighting From Bus Injured In Collision With Two- Or Three-Wheeled Motor Vehicle | Yes | No |
| V72.5 | Driver Of Bus Injured In Collision With Two- Or Three-Wheeled Motor Vehicle In Traffic Accident | Yes | No |
| V72.6 | Passenger On Bus Injured In Collision With Two- Or Three-Wheeled Motor Vehicle In Traffic Accident | Yes | No |
| V72.7 | Person On Outside Of Bus Injured In Collision With Two- Or Three-Wheeled Motor Vehicle In Traffic Accident | Yes | No |
| V72.9 | Unspecified Occupant Of Bus Injured In Collision With Two- Or Three-Wheeled Motor Vehicle In Traffic Accident | Yes | No |
| V73.0 | Driver Of Bus Injured In Collision With Car, Pick-Up Truck Or Van In Nontraffic Accident | Yes | No |
| V73.1 | Passenger On Bus Injured In Collision With Car, Pick-Up Truck Or Van In Nontraffic Accident | Yes | No |
| V73.2 | Person On Outside Of Bus Injured In Collision With Car, Pick-Up Truck Or Van In Nontraffic Accident | Yes | No |
| V73.3 | Unspecified Occupant Of Bus Injured In Collision With Car, Pick-Up Truck Or Van In Nontraffic Accident | Yes | No |
| V73.4 | Person Boarding Or Alighting From Bus Injured In Collision With Car, Pick-Up Truck Or Van | Yes | No |
| V73.5 | Driver Of Bus Injured In Collision With Car, Pick-Up Truck Or Van In Traffic Accident | Yes | No |
| V73.6 | Passenger On Bus Injured In Collision With Car, Pick-Up Truck Or Van In Traffic Accident | Yes | No |
| V73.7 | Person On Outside Of Bus Injured In Collision With Car, Pick-Up Truck Or Van In Traffic Accident | Yes | No |
| V73.9 | Unspecified Occupant Of Bus Injured In Collision With Car, Pick-Up Truck Or Van In Traffic Accident | Yes | No |
| V74.0 | Driver Of Bus Injured In Collision With Heavy Transport Vehicle Or Bus In Nontraffic Accident | Yes | No |
| V74.1 | Passenger On Bus Injured In Collision With Heavy Transport Vehicle Or Bus In Nontraffic Accident | Yes | No |
| V74.2 | Person On Outside Of Bus Injured In Collision With Heavy Transport Vehicle Or Bus In Nontraffic Accident | Yes | No |
| V74.3 | Unspecified Occupant Of Bus Injured In Collision With Heavy Transport Vehicle Or Bus In Nontraffic Accident | Yes | No |
| V74.4 | Person Boarding Or Alighting From Bus Injured In Collision With Heavy Transport Vehicle Or Bus | Yes | No |
| V74.5 | Driver Of Bus Injured In Collision With Heavy Transport Vehicle Or Bus In Traffic Accident | Yes | No |
| V74.6 | Passenger On Bus Injured In Collision With Heavy Transport Vehicle Or Bus In Traffic Accident | Yes | No |
| V74.7 | Person On Outside Of Bus Injured In Collision With Heavy Transport Vehicle Or Bus In Traffic Accident | Yes | No |
| V74.9 | Unspecified Occupant Of Bus Injured In Collision With Heavy Transport Vehicle Or Bus In Traffic Accident | Yes | No |
| V75.0 | Driver Of Bus Injured In Collision With Railway Train Or Railway Vehicle In Nontraffic Accident | Yes | No |
| V75.1 | Passenger On Bus Injured In Collision With Railway Train Or Railway Vehicle In Nontraffic Accident | Yes | No |
| V75.2 | Person On Outside Of Bus Injured In Collision With Railway Train Or Railway Vehicle In Nontraffic Accident | Yes | No |
| V75.3 | Unspecified Occupant Of Bus Injured In Collision With Railway Train Or Railway Vehicle In Nontraffic Accident | Yes | No |
| V75.4 | Person Boarding Or Alighting From Bus Injured In Collision With Railway Train Or Railway Vehicle | Yes | No |
| V75.5 | Driver Of Bus Injured In Collision With Railway Train Or Railway Vehicle In Traffic Accident | Yes | No |
| V75.6 | Passenger On Bus Injured In Collision With Railway Train Or Railway Vehicle In Traffic Accident | Yes | No |
| V75.7 | Person On Outside Of Bus Injured In Collision With Railway Train Or Railway Vehicle In Traffic Accident | Yes | No |
| V75.9 | Unspecified Occupant Of Bus Injured In Collision With Railway Train Or Railway Vehicle In Traffic Accident | Yes | No |
| V76.0 | Driver Of Bus Injured In Collision With Other Nonmotor Vehicle In Nontraffic Accident | Yes | No |
| V76.1 | Passenger On Bus Injured In Collision With Other Nonmotor Vehicle In Nontraffic Accident | Yes | No |
| V76.2 | Person On Outside Of Bus Injured In Collision With Other Nonmotor Vehicle In Nontraffic Accident | Yes | No |
| V76.3 | Unspecified Occupant Of Bus Injured In Collision With Other Nonmotor Vehicle In Nontraffic Accident | Yes | No |
| V76.4 | Person Boarding Or Alighting From Bus Injured In Collision With Other Nonmotor Vehicle | Yes | No |
| V76.5 | Driver Of Bus Injured In Collision With Other Nonmotor Vehicle In Traffic Accident | Yes | No |
| V76.6 | Passenger On Bus Injured In Collision With Other Nonmotor Vehicle In Traffic Accident | Yes | No |
| V76.7 | Person On Outside Of Bus Injured In Collision With Other Nonmotor Vehicle In Traffic Accident | Yes | No |
| V76.9 | Unspecified Occupant Of Bus Injured In Collision With Other Nonmotor Vehicle In Traffic Accident | Yes | No |
| V77.0 | Driver Of Bus Injured In Collision With Fixed Or Stationary Object In Nontraffic Accident | Yes | No |
| V77.1 | Passenger On Bus Injured In Collision With Fixed Or Stationary Object In Nontraffic Accident | Yes | No |
| V77.2 | Person On Outside Of Bus Injured In Collision With Fixed Or Stationary Object In Nontraffic Accident | Yes | No |
| V77.3 | Unspecified Occupant Of Bus Injured In Collision With Fixed Or Stationary Object In Nontraffic Accident | Yes | No |
| V77.4 | Person Boarding Or Alighting From Bus Injured In Collision With Fixed Or Stationary Object | Yes | No |
| V77.5 | Driver Of Bus Injured In Collision With Fixed Or Stationary Object In Traffic Accident | Yes | No |
| V77.6 | Passenger On Bus Injured In Collision With Fixed Or Stationary Object In Traffic Accident | Yes | No |
| V77.7 | Person On Outside Of Bus Injured In Collision With Fixed Or Stationary Object In Traffic Accident | Yes | No |
| V77.9 | Unspecified Occupant Of Bus Injured In Collision With Fixed Or Stationary Object In Traffic Accident | Yes | No |
| V78.0 | Driver Of Bus Injured In Noncollision Transport Accident In Nontraffic Accident | Yes | No |
| V78.1 | Passenger On Bus Injured In Noncollision Transport Accident In Nontraffic Accident | Yes | No |
| V78.2 | Person On Outside Of Bus Injured In Noncollision Transport Accident In Nontraffic Accident | Yes | No |
| V78.3 | Unspecified Occupant Of Bus Injured In Noncollision Transport Accident In Nontraffic Accident | Yes | No |
| V78.4 | Person Boarding Or Alighting From Bus Injured In Noncollision Transport Accident | Yes | No |
| V78.5 | Driver Of Bus Injured In Noncollision Transport Accident In Traffic Accident | Yes | No |
| V78.6 | Passenger On Bus Injured In Noncollision Transport Accident In Traffic Accident | Yes | No |
| V78.7 | Person On Outside Of Bus Injured In Noncollision Transport Accident In Traffic Accident | Yes | No |
| V78.9 | Unspecified Occupant Of Bus Injured In Noncollision Transport Accident In Traffic Accident | Yes | No |
| V79.0 | Driver Of Bus Injured In Collision With Other And Unspecified Motor Vehicles In Nontraffic Accident | Yes | No |
| V79.1 | Passenger On Bus Injured In Collision With Other And Unspecified Motor Vehicles In Nontraffic Accident | Yes | No |
| V79.2 | Unspecified Bus Occupant Injured In Collision With Other And Unspecified Motor Vehicles In Nontraffic Accident | Yes | No |
| V79.3 | Bus Occupant (Driver) (Passenger) Injured In Unspecified Nontraffic Accident | Yes | No |
| V79.4 | Driver Of Bus Injured In Collision With Other And Unspecified Motor Vehicles In Traffic Accident | Yes | No |
| V79.5 | Passenger On Bus Injured In Collision With Other And Unspecified Motor Vehicles In Traffic Accident | Yes | No |
| V79.6 | Unspecified Bus Occupant Injured In Collision With Other And Unspecified Motor Vehicles In Traffic Accident | Yes | No |
| V79.8 | Bus Occupant (Driver) (Passenger) Injured In Other Specified Transport Accidents | Yes | No |
| V79.9 | Bus Occupant (Driver) (Passenger) Injured In Unspecified Traffic Accident | Yes | No |
| V80.0 | Animal-Rider Or Occupant Of Animal Drawn Vehicle Injured By Fall From Or Being Thrown From Animal Or Animal-Drawn Vehicle In Noncollision Accident | No | No |
| V80.1 | Animal-Rider Or Occupant Of Animal-Drawn Vehicle Injured In Collision With Pedestrian Or Animal | No | No |
| V80.2 | Animal-Rider Or Occupant Of Animal-Drawn Vehicle Injured In Collision With Pedal Cycle | No | No |
| V80.3 | Animal-Rider Or Occupant Of Animal-Drawn Vehicle Injured In Collision With Two- Or Three-Wheeled Motor Vehicle | Yes | No |
| V80.4 | Animal-Rider Or Occupant Of Animal-Drawn Vehicle Injured In Collision With Car, Pick-Up Truck, Van, Heavy Transport Vehicle Or Bus | Yes | No |
| V80.5 | Animal-Rider Or Occupant Of Animal-Drawn Vehicle Injured In Collision With Other Specified Motor Vehicle | Yes | No |
| V80.6 | Animal-Rider Or Occupant Of Animal-Drawn Vehicle Injured In Collision With Railway Train Or Railway Vehicle | No | No |
| V80.7 | Animal-Rider Or Occupant Of Animal-Drawn Vehicle Injured In Collision With Other Nonmotor Vehicles | No | No |
| V80.8 | Animal-Rider Or Occupant Of Animal-Drawn Vehicle Injured In Collision With Fixed Or Stationary Object | No | No |
| V80.9 | Animal-Rider Or Occupant Of Animal-Drawn Vehicle Injured In Other And Unspecified Transport Accidents | No | No |
| V81.0 | Occupant Of Railway Train Or Railway Vehicle Injured In Collision With Motor Vehicle In Nontraffic Accident | Yes | No |
| V81.1 | Occupant Of Railway Train Or Railway Vehicle Injured In Collision With Motor Vehicle In Traffic Accident | Yes | No |
| V81.2 | Occupant Of Railway Train Or Railway Vehicle Injured In Collision With Or Hit By Rolling Stock | No | No |
| V81.3 | Occupant Of Railway Train Or Railway Vehicle Injured In Collision With Other Object | No | No |
| V81.4 | Person Injured While Boarding Or Alighting From Railway Train Or Railway Vehicle | No | No |
| V81.5 | Occupant Of Railway Train Or Railway Vehicle Injured By Fall In Railway Train Or Railway Vehicle | No | No |
| V81.6 | Occupant Of Railway Train Or Railway Vehicle Injured By Fall From Railway Train Or Railway Vehicle | No | No |
| V81.7 | Occupant Of Railway Train Or Railway Vehicle Injured In Derailment Without Antecedent Collision | No | No |
| V81.8 | Occupant Of Railway Train Or Railway Vehicle Injured In Other Specified Railway Accidents | No | No |
| V81.9 | Occupant Of Railway Train Or Railway Vehicle Injured In Unspecified Railway Accident | No | No |
| V82.0 | Occupant Of Streetcar Injured In Collision With Motor Vehicle In Nontraffic Accident | Yes | No |
| V82.1 | Occupant Of Streetcar Injured In Collision With Motor Vehicle In Traffic Accident | Yes | No |
| V82.2 | Occupant Of Streetcar Injured In Collision With Or Hit By Rolling Stock | No | No |
| V82.3 | Occupant Of Streetcar Injured In Collision With Other Object | No | No |
| V82.4 | Person Injured While Boarding Or Alighting From Streetcar | No | No |
| V82.5 | Occupant Of Streetcar Injured By Fall In Streetcar | No | No |
| V82.6 | Occupant Of Streetcar Injured By Fall From Streetcar | No | No |
| V82.7 | Occupant Of Streetcar Injured In Derailment Without Antecedent Collision | No | No |
| V82.8 | Occupant Of Streetcar Injured In Other Specified Transport Accidents | No | No |
| V82.9 | Occupant Of Streetcar Injured In Unspecified Traffic Accident | No | No |
| V83.0 | Driver Of Special Industrial Vehicle Injured In Traffic Accident | Yes | No |
| V83.1 | Passenger Of Special Industrial Vehicle Injured In Traffic Accident | Yes | No |
| V83.2 | Person On Outside Of Special Industrial Vehicle Injured In Traffic Accident | Yes | No |
| V83.3 | Unspecified Occupant Of Special Industrial Vehicle Injured In Traffic Accident | Yes | No |
| V83.4 | Person Injured While Boarding Or Alighting From Special Industrial Vehicle | Yes | No |
| V83.5 | Driver Of Special Industrial Vehicle Injured In Nontraffic Accident | Yes | No |
| V83.6 | Passenger Of Special Industrial Vehicle Injured In Nontraffic Accident | Yes | No |
| V83.7 | Person On Outside Of Special Industrial Vehicle Injured In Nontraffic Accident | Yes | No |
| V83.9 | Unspecified Occupant Of Special Industrial Vehicle Injured In Nontraffic Accident | Yes | No |
| V84.0 | Driver Of Special Agricultural Vehicle Injured In Traffic Accident | Yes | No |
| V84.1 | Passenger Of Special Agricultural Vehicle Injured In Traffic Accident | Yes | No |
| V84.2 | Person On Outside Of Special Agricultural Vehicle Injured In Traffic Accident | Yes | No |
| V84.3 | Unspecified Occupant Of Special Agricultural Vehicle Injured In Traffic Accident | Yes | No |
| V84.4 | Person Injured While Boarding Or Alighting From Special Agricultural Vehicle | Yes | No |
| V84.5 | Driver Of Special Agricultural Vehicle Injured In Nontraffic Accident | Yes | No |
| V84.6 | Passenger Of Special Agricultural Vehicle Injured In Nontraffic Accident | Yes | No |
| V84.7 | Person On Outside Of Special Agricultural Vehicle Injured In Nontraffic Accident | Yes | No |
| V84.9 | Unspecified Occupant Of Special Agricultural Vehicle Injured In Nontraffic Accident | Yes | No |
| V85.0 | Driver Of Special Construction Vehicle Injured In Traffic Accident | Yes | No |
| V85.1 | Passenger Of Special Construction Vehicle Injured In Traffic Accident | Yes | No |
| V85.2 | Person On Outside Of Special Construction Vehicle Injured In Traffic Accident | Yes | No |
| V85.3 | Unspecified Occupant Of Special Construction Vehicle Injured In Traffic Accident | Yes | No |
| V85.4 | Person Injured While Boarding Or Alighting From Special Construction Vehicle | Yes | No |
| V85.5 | Driver Of Special Construction Vehicle Injured In Nontraffic Accident | Yes | No |
| V85.6 | Passenger Of Special Construction Vehicle Injured In Nontraffic Accident | Yes | No |
| V85.7 | Person On Outside Of Special Construction Vehicle Injured In Nontraffic Accident | Yes | No |
| V85.9 | Unspecified Occupant Of Special Construction Vehicle Injured In Nontraffic Accident | Yes | No |
| V86.0 | Driver Of Special All-Terrain Or Other Off-Road Motor Vehicle Injured In Traffic Accident | Yes | No |
| V86.1 | Passenger Of Special All-Terrain Or Other Off-Road Motor Vehicle Injured In Traffic Accident | Yes | No |
| V86.2 | Person On Outside Of Special All-Terrain Or Other Off-Road Motor Vehicle Injured In Traffic Accident | Yes | No |
| V86.3 | Unspecified Occupant Of Special All-Terrain Or Other Off-Road Motor Vehicle Injured In Traffic Accident | Yes | No |
| V86.4 | Person Injured While Boarding Or Alighting From Special All-Terrain Or Other Off-Road Motor Vehicle | Yes | No |
| V86.5 | Driver Of Special All-Terrain Or Other Off-Road Motor Vehicle Injured In Nontraffic Accident | Yes | No |
| V86.6 | Passenger Of Special All-Terrain Or Other Off-Road Motor Vehicle Injured In Nontraffic Accident | Yes | No |
| V86.7 | Person On Outside Of Special All-Terrain Or Other Off-Road Motor Vehicle Injured In Nontraffic Accident | Yes | No |
| V86.9 | Unspecified Occupant Of Special All-Terrain Or Other Off-Road Motor Vehicle Injured In Nontraffic Accident | Yes | No |
| V87.0 | Person Injured In Collision Between Car And Two- Or Three-Wheeled Powered Vehicle (Traffic) | Yes | No |
| V87.1 | Person Injured In Collision Between Other Motor Vehicle And Two- Or Three-Wheeled Motor Vehicle (Traffic) | Yes | No |
| V87.2 | Person Injured In Collision Between Car And Pick-Up Truck Or Van (Traffic) | Yes | No |
| V87.3 | Person Injured In Collision Between Car And Bus (Traffic) | Yes | No |
| V87.4 | Person Injured In Collision Between Car And Heavy Transport Vehicle (Traffic) | Yes | No |
| V87.5 | Person Injured In Collision Between Heavy Transport Vehicle And Bus (Traffic) | Yes | No |
| V87.6 | Person Injured In Collision Between Railway Train Or Railway Vehicle And Car (Traffic) | Yes | No |
| V87.7 | Person Injured In Collision Between Other Specified Motor Vehicles (Traffic) | Yes | No |
| V87.8 | Person Injured In Other Specified Noncollision Transport Accidents Involving Motor Vehicle (Traffic) | Yes | No |
| V87.9 | Person Injured In Other Specified (Collision)(Noncollision) Transport Accidents Involving Nonmotor Vehicle (Traffic) | No | No |
| V88.0 | Person Injured In Collision Between Car And Two- Or Three-Wheeled Motor Vehicle, Nontraffic | Yes | No |
| V88.1 | Person Injured In Collision Between Other Motor Vehicle And Two- Or Three-Wheeled Motor Vehicle, Nontraffic | Yes | No |
| V88.2 | Person Injured In Collision Between Car And Pick-Up Truck Or Van, Nontraffic | Yes | No |
| V88.3 | Person Injured In Collision Between Car And Bus, Nontraffic | Yes | No |
| V88.4 | Person Injured In Collision Between Car And Heavy Transport Vehicle, Nontraffic | Yes | No |
| V88.5 | Person Injured In Collision Between Heavy Transport Vehicle And Bus, Nontraffic | Yes | No |
| V88.6 | Person Injured In Collision Between Railway Train Or Railway Vehicle And Car, Nontraffic | Yes | No |
| V88.7 | Person Injured In Collision Between Other Specified Motor Vehicle, Nontraffic | Yes | No |
| V88.8 | Person Injured In Other Specified Noncollision Transport Accidents Involving Motor Vehicle, Nontraffic | Yes | No |
| V88.9 | Person Injured In Other Specified (Collision)(Noncollision) Transport Accidents Involving Nonmotor Vehicle, Nontraffic | No | No |
| V89.0 | Person Injured In Unspecified Motor-Vehicle Accident, Nontraffic | Yes | No |
| V89.1 | Person Injured In Unspecified Nonmotor-Vehicle Accident, Nontraffic | No | No |
| V89.2 | Person Injured In Unspecified Motor-Vehicle Accident, Traffic | Yes | No |
| V89.3 | Person Injured In Unspecified Nonmotor-Vehicle Accident, Traffic | No | No |
| V89.9 | Person Injured In Unspecified Vehicle Accident | No | No |
| V90.0 | Drowning And Submersion Due To Watercraft Overturning | No | No |
| V90.1 | Drowning And Submersion Due To Watercraft Sinking | No | No |
| V90.2 | Drowning And Submersion Due To Falling Or Jumping From Burning Watercraft | No | No |
| V90.3 | Drowning And Submersion Due To Falling Or Jumping From Crushed Watercraft | No | No |
| V90.4 | ***PARENT CODE = Drowning And Submersion Due To Accident To Watercraft *** | No | No |
| V90.5 | ***PARENT CODE = Drowning And Submersion Due To Accident To Watercraft *** | No | No |
| V90.6 | ***PARENT CODE = Drowning And Submersion Due To Accident To Watercraft *** | No | No |
| V90.7 | ***PARENT CODE = Drowning And Submersion Due To Accident To Watercraft *** | No | No |
| V90.8 | Drowning And Submersion Due To Other Accident To Watercraft | No | No |
| V90.9 | ***PARENT CODE = Drowning And Submersion Due To Accident To Watercraft *** | No | No |
| V91.0 | Burn Due To Watercraft On Fire | No | No |
| V91.1 | Crushed Between Watercraft And Other Watercraft Or Other Object Due To Collision | No | No |
| V91.2 | Fall Due To Collision Between Watercraft And Other Watercraft Or Other Object | No | No |
| V91.3 | Hit Or Struck By Falling Object Due To Accident To Watercraft | No | No |
| V91.4 | ***PARENT CODE = Other Injury Due To Accident To Watercraft *** | No | No |
| V91.5 | ***PARENT CODE = Other Injury Due To Accident To Watercraft *** | No | No |
| V91.6 | ***PARENT CODE = Other Injury Due To Accident To Watercraft *** | No | No |
| V91.7 | ***PARENT CODE = Other Injury Due To Accident To Watercraft *** | No | No |
| V91.8 | Other Injury Due To Other Accident To Watercraft | No | No |
| V91.9 | ***PARENT CODE = Other Injury Due To Accident To Watercraft *** | No | No |
| V92.0 | Drowning And Submersion Due To Fall Off Watercraft | No | No |
| V92.1 | Drowning And Submersion Due To Being Thrown Overboard By Motion Of Watercraft | No | No |
| V92.2 | Drowning And Submersion Due To Being Washed Overboard From Watercraft | No | No |
| V92.3 | ***PARENT CODE = Drowning And Submersion Due To Accident On Board Watercraft, Without Accident To Watercraft *** | No | No |
| V92.4 | ***PARENT CODE = Drowning And Submersion Due To Accident On Board Watercraft, Without Accident To Watercraft *** | No | No |
| V92.5 | ***PARENT CODE = Drowning And Submersion Due To Accident On Board Watercraft, Without Accident To Watercraft *** | No | No |
| V92.6 | ***PARENT CODE = Drowning And Submersion Due To Accident On Board Watercraft, Without Accident To Watercraft *** | No | No |
| V92.7 | ***PARENT CODE = Drowning And Submersion Due To Accident On Board Watercraft, Without Accident To Watercraft *** | No | No |
| V92.8 | ***PARENT CODE = Drowning And Submersion Due To Accident On Board Watercraft, Without Accident To Watercraft *** | No | No |
| V92.9 | ***PARENT CODE = Drowning And Submersion Due To Accident On Board Watercraft, Without Accident To Watercraft *** | No | No |
| V93.0 | Burn Due To Localized Fire On Board Watercraft | No | No |
| V93.1 | Other Burn On Board Watercraft | No | No |
| V93.2 | Heat Exposure On Board Watercraft | No | No |
| V93.3 | Fall On Board Watercraft | No | No |
| V93.4 | Struck By Falling Object On Board Watercraft | No | No |
| V93.5 | Explosion On Board Watercraft | No | No |
| V93.6 | Machinery Accident On Board Watercraft | No | No |
| V93.7 | ***PARENT CODE = Other Injury Due To Accident On Board Watercraft, Without Accident To Watercraft *** | No | No |
| V93.8 | Other Injury Due To Other Accident On Board Watercraft | No | No |
| V93.9 | ***PARENT CODE = Other Injury Due To Accident On Board Watercraft, Without Accident To Watercraft *** | No | No |
| V94.0 | Hitting Object Or Bottom Of Body Of Water Due To Fall From Watercraft | No | No |
| V94.1 | Bather Struck By Watercraft | No | No |
| V94.2 | Rider Of Nonpowered Watercraft Struck By Other Watercraft | No | No |
| V94.3 | Injury To Rider Of (Inflatable) Watercraft Being Pulled Behind Other Watercraft | No | No |
| V94.4 | Injury To Barefoot Water-Skier | No | No |
| V94.5 | ***PARENT CODE = Other And Unspecified Water Transport Accidents *** | No | No |
| V94.6 | ***PARENT CODE = Other And Unspecified Water Transport Accidents *** | No | No |
| V94.7 | ***PARENT CODE = Other And Unspecified Water Transport Accidents *** | No | No |
| V94.8 | Other Water Transport Accident | No | No |
| V94.9 | Unspecified Water Transport Accident | No | No |
| V95.0 | Helicopter Accident Injuring Occupant | No | No |
| V95.1 | Ultralight, Microlight Or Powered-Glider Accident Injuring Occupant | No | No |
| V95.2 | Other Private Fixed-Wing Aircraft Accident Injuring Occupant | No | No |
| V95.3 | Commercial Fixed-Wing Aircraft Accident Injuring Occupant | No | No |
| V95.4 | Spacecraft Accident Injuring Occupant | No | No |
| V95.8 | Other Powered Aircraft Accidents Injuring Occupant | No | No |
| V95.9 | Unspecified Aircraft Accident Injuring Occupant | No | No |
| V96.0 | Balloon Accident Injuring Occupant | No | No |
| V96.1 | Hang-Glider Accident Injuring Occupant | No | No |
| V96.2 | Glider (Nonpowered) Accident Injuring Occupant | No | No |
| V96.8 | Other Nonpowered-Aircraft Accidents Injuring Occupant | No | No |
| V96.9 | Unspecified Nonpowered-Aircraft Accident Injuring Occupant | No | No |
| V97.0 | Occupant Of Aircraft Injured In Other Specified Air Transport Accidents | No | No |
| V97.1 | Person Injured While Boarding Or Alighting From Aircraft | No | No |
| V97.2 | Parachutist Accident | No | No |
| V97.3 | Person On Ground Injured In Air Transport Accident | No | No |
| V97.8 | Other Air Transport Accidents, Not Elsewhere Classified | No | No |
| V98. | Other Specified Transport Accidents | No | No |
| V99. | Unspecified Transport Accident | No | No |
| W00. | Fall Due To Ice And Snow | No | No |
| W01. | Fall On Same Level From Slipping, Tripping And Stumbling | No | No |
| W02. | Fall involving ice-skates, skis, roller-skates or skateboards | No | No |
| W03. | Other Fall On Same Level Due To Collision With Another Person | No | No |
| W04. | Fall While Being Carried Or Supported By Other Persons | No | No |
| W05. | Fall From Non-Moving Wheelchair, Nonmotorized Scooter And Motorized Mobility Scooter | No | No |
| W06. | Fall From Bed | No | No |
| W07. | Fall From Chair | No | No |
| W08. | Fall From Other Furniture | No | No |
| W09. | Fall On And From Playground Equipment | No | No |
| W10. | Fall On And From Stairs And Steps | No | No |
| W11. | Fall On And From Ladder | No | No |
| W12. | Fall On And From Scaffolding | No | No |
| W13. | Fall From, Out Of Or Through Building Or Structure | No | No |
| W14. | Fall From Tree | No | No |
| W15. | Fall From Cliff | No | No |
| W16. | Fall, Jump Or Diving Into Water | No | No |
| W17. | Other Fall From One Level To Another | No | No |
| W18. | Other Slipping, Tripping And Stumbling And Falls | No | No |
| W19. | Unspecified Fall | No | No |
| W20. | Struck By Thrown, Projected Or Falling Object | No | No |
| W21. | Striking Against Or Struck By Sports Equipment | No | No |
| W22. | Striking Against Or Struck By Other Objects | No | No |
| W23. | Caught, Crushed, Jammed Or Pinched In Or Between Objects | No | No |
| W24. | Contact With Lifting And Transmission Devices, Not Elsewhere Classified | No | No |
| W25. | Contact With Sharp Glass | No | No |
| W26. | Contact With Knife, Sword Or Dagger | No | No |
| W27. | Contact With Nonpowered Hand Tool | No | No |
| W28. | Contact With Powered Lawn Mower | No | No |
| W29. | Contact With Other Powered Hand Tools And Household Machinery | No | No |
| W30. | Contact With Agricultural Machinery | No | No |
| W31. | Contact With Other And Unspecified Machinery | No | No |
| W32. | Accidental Handgun Discharge And Malfunction | No | No |
| W33. | Accidental Rifle, Shotgun And Larger Firearm Discharge And Malfunction | No | No |
| W34. | Accidental Discharge And Malfunction From Other And Unspecified Firearms And Guns | No | No |
| W35. | Explosion And Rupture Of Boiler | No | No |
| W36. | Explosion And Rupture Of Gas Cylinder | No | No |
| W37. | Explosion And Rupture Of Pressurized Tire, Pipe Or Hose | No | No |
| W38. | Explosion And Rupture Of Other Specified Pressurized Devices | No | No |
| W39. | Discharge Of Firework | No | No |
| W40. | Explosion Of Other Materials | No | No |
| W41. | Exposure to high-pressure jet | No | No |
| W42. | Exposure To Noise | No | No |
| W43. | Exposure to vibration | No | No |
| W44. | Foreign body entering into or through eye or natural orifice | No | No |
| W45. | Foreign Body Or Object Entering Through Skin | No | No |
| W49. | Exposure To Other Inanimate Mechanical Forces | No | No |
| W50. | Accidental Hit, Strike, Kick, Twist, Bite Or Scratch By Another Person | No | No |
| W51. | Accidental Striking Against Or Bumped Into By Another Person | No | No |
| W52. | Crushed, Pushed Or Stepped On By Crowd Or Human Stampede | No | No |
| W53. | Contact With Rodent | No | No |
| W54. | Contact With Dog | No | No |
| W55. | Contact With Other Mammals | No | No |
| W56. | Contact With Nonvenomous Marine Animal | No | No |
| W57. | Bitten Or Stung By Nonvenomous Insect And Other Nonvenomous Arthropods | No | No |
| W58. | Contact With Crocodile Or Alligator | No | No |
| W59. | Contact With Other Nonvenomous Reptiles | No | No |
| W60. | Contact With Nonvenomous Plant Thorns And Spines And Sharp Leaves | No | No |
| W64. | Exposure To Other Animate Mechanical Forces | No | No |
| W65. | Accidental Drowning And Submersion While In Bath-Tub | No | No |
| W67. | Accidental Drowning And Submersion While In Swimming-Pool | No | No |
| W68. | Drowning and submersion following fall into swimming-pool | No | No |
| W69. | Accidental Drowning And Submersion While In Natural Water | No | No |
| W70. | Drowning and submersion following fall into natural water | No | No |
| W73. | Other Specified Cause Of Accidental Non-Transport Drowning And Submersion | No | No |
| W74. | Unspecified Cause Of Accidental Drowning And Submersion | No | No |
| W75. | Accidental suffocation and strangulation in bed | No | No |
| W76. | Other accidental hanging and strangulation | No | No |
| W77. | Threat to breathing due to cave-in, falling earth and other substances | No | No |
| W78. | Inhalation of gastric contents | No | No |
| W79. | Inhalation and ingestion of food causing obstruction of respiratory tract | No | No |
| W80. | Inhalation and ingestion of other objects causing obstruction of respiratory tract | No | No |
| W81. | Confined to or trapped in a low-oxygen environment | No | No |
| W83. | Other specified threats to breathing | No | No |
| W84. | Unspecified threat to breathing | No | No |
| W85. | Exposure To Electric Transmission Lines | No | No |
| W86. | Exposure To Other Specified Electric Current | No | No |
| W87. | Exposure to unspecified electric current | No | No |
| W88. | Exposure To Ionizing Radiation | No | No |
| W89. | Exposure To Man-Made Visible And Ultraviolet Light | No | No |
| W90. | Exposure To Other Nonionizing Radiation | No | No |
| W91. | Exposure to unspecified type of radiation | No | No |
| W92. | Exposure To Excessive Heat Of Man-Made Origin | No | No |
| W93. | Exposure To Excessive Cold Of Man-Made Origin | No | No |
| W94. | Exposure To High And Low Air Pressure And Changes In Air Pressure | No | No |
| W99. | Exposure To Other Man-Made Environmental Factors | No | No |
| X00. | Exposure To Uncontrolled Fire In Building Or Structure | No | No |
| X01. | Exposure To Uncontrolled Fire, Not In Building Or Structure | No | No |
| X02. | Exposure To Controlled Fire In Building Or Structure | No | No |
| X03. | Exposure To Controlled Fire, Not In Building Or Structure | No | No |
| X04. | Exposure To Ignition Of Highly Flammable Material | No | No |
| X05. | Exposure To Ignition Or Melting Of Nightwear | No | No |
| X06. | Exposure To Ignition Or Melting Of Other Clothing And Apparel | No | No |
| X08. | Exposure To Other Specified Smoke, Fire And Flames | No | No |
| X09. | Exposure to unspecified smoke, fire and flames | No | No |
| X10. | Contact With Hot Drinks, Food, Fats And Cooking Oils | No | No |
| X11. | Contact With Hot Tap-Water | No | No |
| X12. | Contact With Other Hot Fluids | No | No |
| X13. | Contact With Steam And Other Hot Vapors | No | No |
| X14. | Contact With Hot Air And Other Hot Gases | No | No |
| X15. | Contact With Hot Household Appliances | No | No |
| X16. | Contact With Hot Heating Appliances, Radiators And Pipes | No | No |
| X17. | Contact With Hot Engines, Machinery And Tools | No | No |
| X18. | Contact With Other Hot Metals | No | No |
| X19. | Contact With Other Heat And Hot Substances | No | No |
| X20. | Contact with venomous snakes and lizards | No | No |
| X21. | Contact with venomous spiders | No | No |
| X22. | Contact with scorpions | No | No |
| X23. | Contact with hornets, wasps and bees | No | No |
| X24. | Contact with centipedes and venomous millipedes (tropical) | No | No |
| X25. | Contact with other venomous arthropods | No | No |
| X26. | Contact with venomous marine animals and plants | No | No |
| X27. | Contact with other specified venomous animals | No | No |
| X28. | Contact with other specified venomous plants | No | No |
| X29. | Contact with unspecified venomous animal or plant | No | No |
| X30. | Exposure To Excessive Natural Heat | No | No |
| X31. | Exposure To Excessive Natural Cold | No | No |
| X32. | Exposure To Sunlight | No | No |
| X33. | Victim of lightning | No | No |
| X34. | Earthquake | No | No |
| X35. | Volcanic Eruption | No | No |
| X36. | Avalanche, Landslide And Other Earth Movements | No | No |
| X37. | Cataclysmic Storm | No | No |
| X38. | Flood | No | No |
| X39. | Exposure To Other Forces Of Nature | No | No |
| X39.8 | Other Exposure To Forces Of Nature | No | No |
| X40. | Accidental poisoning by and exposure to nonopioid analgesics, antipyretics and antirheumatics | No | Yes |
| X41. | Accidental poisoning by and exposure to antiepileptic, sedative-hypnotic, antiparkinsonism and psychotropic drugs, not elsewhere classified | No | Yes |
| X42. | Accidental poisoning by and exposure to narcotics and psychodysleptics [hallucinogens], not elsewhere classified | No | Yes |
| X43. | Accidental poisoning by and exposure to other drugs acting on the autonomic nervous system | No | Yes |
| X44. | Accidental poisoning by and exposure to other and unspecified drugs, medicaments and biological substances | No | Yes |
| X45. | Accidental poisoning by and exposure to alcohol | No | Yes |
| X46. | Accidental poisoning by and exposure to organic solvents and halogenated hydrocarbons and their vapours | No | No |
| X47. | Accidental poisoning by and exposure to carbon monoxide and other gases and vapours | No | No |
| X48. | Accidental poisoning by and exposure to pesticides | No | No |
| X49. | Accidental poisoning by and exposure to other and unspecified chemicals and noxious substances | No | No |
| X52. | Prolonged Stay In Weightless Environment | No | No |
| X53. | Lack of food | No | No |
| X54. | Lack of water | No | No |
| X57. | Unspecified privation | No | No |
| X58. | Exposure To Other Specified Factors | No | No |
| X59. | Exposure to unspecified factor | No | No |
| Y85.0 | Sequelae of motor-vehicle accident | No | No |
| Y85.9 | Sequelae of other and unspecified transport accidents | No | No |
| Y86. | Sequelae of other accidents | No | No |
